# Genome-wide association study of thoracic aortic aneurysm and dissection in the Million Veteran Program

**DOI:** 10.1038/s41588-023-01420-z

**Published:** 2023-06-12

**Authors:** Derek Klarin, Poornima Devineni, Anoop K. Sendamarai, Anthony R. Angueira, Sarah E. Graham, Ying H. Shen, Michael G. Levin, James P. Pirruccello, Ida Surakka, Purushotham R. Karnam, Tanmoy Roychowdhury, Yanming Li, Minxian Wang, Krishna G. Aragam, Kaavya Paruchuri, Verena Zuber, Gabrielle E. Shakt, Noah L. Tsao, Renae L. Judy, Ha My T. Vy, Shefali S. Verma, Daniel J. Rader, Ron Do, Joseph E. Bavaria, Girish N. Nadkarni, Marylyn D. Ritchie, VA Million Veteran Program, Stephen Burgess, Dong-chuan Guo, Patrick T. Ellinor, Scott A. LeMaire, Dianna M. Milewicz, Cristen J. Willer, Pradeep Natarajan, Philip S. Tsao, Saiju Pyarajan, Scott M. Damrauer

**Affiliations:** 1 Veterans Affairs (VA) Palo Alto Healthcare System, Palo Alto, CA USA; 2 Department of Surgery, Stanford University School of Medicine, Palo Alto, CA USA; 3 Center for Data and Computational Sciences, VA Boston Healthcare System, Boston, MA USA; 4 Carbone Cancer Center, University of Wisconsin—Madison, Madison, WI USA; 5 Department of Medicine, Harvard Medical School, Boston, MA USA; 6 Institute for Diabetes, Obesity and Metabolism, Perelman School of Medicine at the University of Pennsylvania, Philadelphia, PA USA; 7 Department of Internal Medicine, Division of Cardiology, University of Michigan, Ann Arbor, MI USA; 8 Division of Cardiothoracic Surgery, Michael E. DeBakey Department of Surgery, Baylor College of Medicine, Houston, TX USA; 9 Department of Cardiovascular Surgery, Texas Heart Institute, Houston, TX USA; 10 Division of Cardiovascular Medicine, Department of Medicine, University of Pennsylvania Perelman School of Medicine, Philadelphia, PA USA; 11 Department of Medicine, Corporal Michael J. Crescenz VA Medical Center, Philadelphia, PA USA; 12 Division of Cardiology, University of California San Francisco, San Francisco, CA USA; 13 Institute for Human Genetics, University of California San Francisco, San Francisco, CA USA; 14 Department of Genetics, Yale School of Medicine, New Haven, CT USA; 15 CAS Key Laboratory of Genome Sciences and Information, Beijing Institute of Genomics, Chinese Academy of Sciences and China National Center for Bioinformation, Beijing, China; 16 University of Chinese Academy of Sciences, Beijing, China; 17 Cardiovascular Research Center, Massachusetts General Hospital, Boston, MA USA; 18 Program in Medical and Population Genetics, Broad Institute of MIT and Harvard, Cambridge, MA USA; 19 Cardiovascular Disease Initiative, Broad Institute of MIT and Harvard, Cambridge, MA USA; 20 Department of Epidemiology and Biostatistics, School of Public Health, Imperial College London, London, UK; 21 MRC Centre for Environment and Health, School of Public Health, Imperial College London, London, UK; 22 UK Dementia Research Institute at Imperial College, Imperial College London, London, UK; 23 Department of Surgery, Perelman School of Medicine, University of Pennsylvania, Philadelphia, USA; 24 Icahn School of Medicine at Mount Sinai, New York, NY USA; 25 Department of Pathology and Laboratory Medicine, Perelman School of Medicine, University of Pennsylvania, Philadelphia, PA USA; 26 Division of Translational Medicine and Human Genetics, Department of Medicine, Perelman School of Medicine at the University of Pennsylvania, Philadelphia, PA USA; 27 Department of Genetics, Perelman School of Medicine at the University of Pennsylvania, Philadelphia, PA USA; 28 Division of Cardiovascular Surgery, Perelman School of Medicine, University of Pennsylvania, Philadelphia, PA USA; 29 Department of Genetics, Perelman School of Medicine, University of Pennsylvania, Philadelphia, PA USA; 30 Institute for Biomedical Informatics, University of Pennsylvania, Perelman School of Medicine, Philadelphia, PA USA; 31 Medical Research Council Biostatistics Unit, University of Cambridge, Cambridge, UK; 32 Cardiovascular Epidemiology Unit, Department of Public Health and Primary Care, University of Cambridge, Cambridge, UK; 33 Division of Medical Genetics, Department of Internal Medicine, McGovern Medical School, University of Texas Health Science Center at Houston, Houston, TX USA; 34 Cardiology Division, Massachusetts General Hospital, Boston, MA USA; 35 Cardiovascular Research Institute, Baylor College of Medicine, Houston, TX USA; 36 Department of Computational Medicine and Bioinformatics, University of Michigan, Ann Arbor, MI USA; 37 Department of Human Genetics, University of Michigan, Ann Arbor, MI USA; 38 Department of Medicine, Stanford University School of Medicine, Stanford, CA USA; 39 Stanford Cardiovascular Institute, Stanford, CA USA; 40 Corporal Michael J. Crescenz VA Medical Center, Philadelphia, PA USA; 41 Department of Surgery, Perelman School of Medicine, University of Pennsylvania, Philadelphia, PA USA

**Keywords:** Genome-wide association studies, Computational biology and bioinformatics

## Abstract

The current understanding of the genetic determinants of thoracic aortic aneurysms and dissections (TAAD) has largely been informed through studies of rare, Mendelian forms of disease. Here, we conducted a genome-wide association study (GWAS) of TAAD, testing ~25 million DNA sequence variants in 8,626 participants with and 453,043 participants without TAAD in the Million Veteran Program, with replication in an independent sample of 4,459 individuals with and 512,463 without TAAD from six cohorts. We identified 21 TAAD risk loci, 17 of which have not been previously reported. We leverage multiple downstream analytic methods to identify causal TAAD risk genes and cell types and provide human genetic evidence that TAAD is a non-atherosclerotic aortic disorder distinct from other forms of vascular disease. Our results demonstrate that the genetic architecture of TAAD mirrors that of other complex traits and that it is not solely inherited through protein-altering variants of large effect size.

## Abstract (web summary)

A genome-wide association study of thoracic aortic aneurysms and dissections (TAAD) in the Million Veteran Program identifies 17 new risk loci and demonstrates that the genetic architecture of TAAD mirrors that of other complex traits.

## Main

TAAD encompass a spectrum of aortic pathology affecting the aortic root, the ascending aorta, the aortic arch and the descending thoracic aorta. Thoracic aortic aneurysms, a dilation of the proximal aorta, are known to progressively enlarge over time, ultimately leading to rupture and death if not surgically repaired. In addition, dissections of the ascending (Stanford type A) or descending (Stanford type B) thoracic aorta are life-threatening conditions requiring emergency treatment, often including surgical repair, and are associated with high short-term and long-term mortality risk^1,2^. Despite the lethality of these conditions, the genetic determinants of TAAD remain largely unknown, with published GWAS having revealed only four loci reaching genome-wide significance^3–5^. As a result, most of what is understood about the genetics of TAAD has been derived from studies examining rare, pathogenic variants resulting in heritable aortopathy (so called, hereditary TAAD or ‘HTAAD’)^6^.

**Fig. 1:**
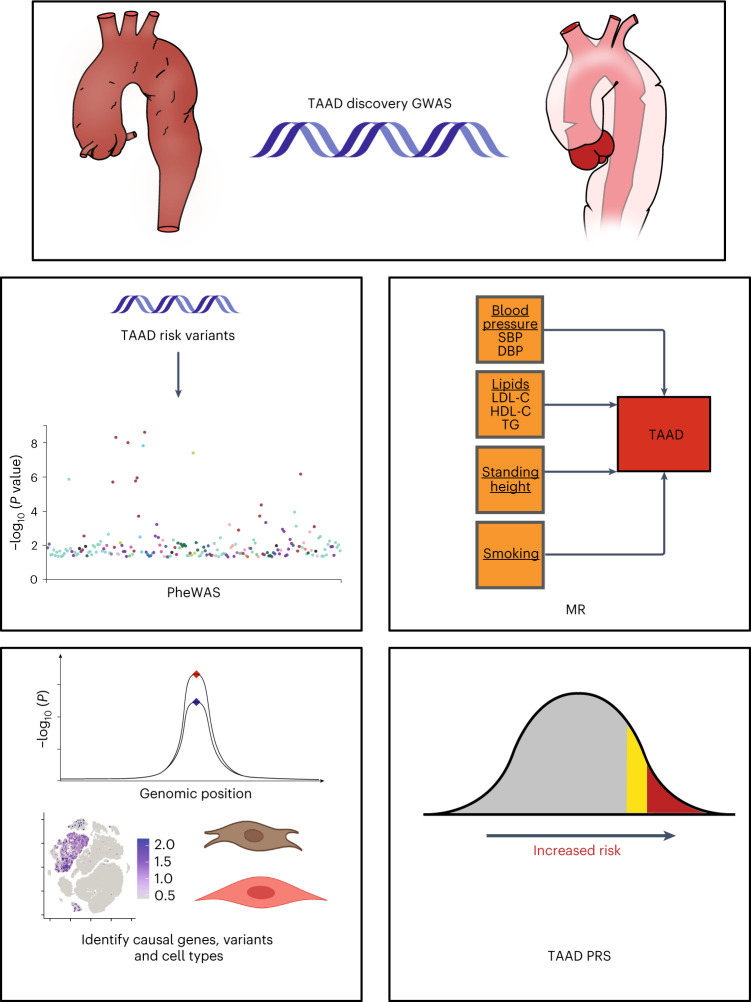
Overall study design. In the current study, we first performed a TAAD discovery GWAS in the MVP, with replication from six external datasets. Secondary analyses included a PheWAS of lead TAAD risk variants, MR analyses with known epidemiologic risk factors for disease, a series of analyses to identify causal genes, variants and cell types for TAAD leveraging colocalization techniques and scRNA-seq or snRNA-seq data, and the generation and testing of a TAAD PRS. This figure was created with the assistance of BioRender. Abbreviations: LDL-C, LDL cholesterol; HDL-C, HDL cholesterol; TG, triglycerides; SBP, systolic blood pressure; DBP, diastolic blood pressure.

The Million Veteran Program (MVP) is a genomic and precision medicine cohort established in 2011 by the Department of VA Healthcare System to study how genes affect health and disease. We recently demonstrated that a VA Healthcare System-based biobank can aid in the genetic discovery of aortic disease^7^ and allows for the elucidation of causal biology and mechanisms. Leveraging the MVP resource, we sought to (1) perform a genetic discovery analysis for TAAD across multiple ancestries, (2) explore the spectrum of phenotypes associated with TAAD risk variants through a phenome-wide association study (PheWAS), (3) examine the genetic relationship between TAAD and its epidemiologic risk factors, (4) map causal variants and genes for disease, (5) identify causal tissues and cell types and (6) construct and test a polygenic risk score (PRS) for TAAD (Fig. 1).

## Results

### Common variants associated with TAAD

We designed a two-phased GWAS. The initial MVP discovery analysis was composed of 8,626 individuals (7,050 European, 1,266 African and 310 Hispanic ancestry participants) with TAAD and 453,043 disease-free individuals from the same ancestral groups (Supplementary Fig. 1); their baseline characteristics are presented in Supplementary Table 1. Participants with TAAD were more likely to be older, male, prescribed statin therapy and former smokers.

Through genotype imputation, we obtained 25.4 million, 40.3 million and 34.9 million DNA sequence variants for analysis in participants of European, African and Hispanic ancestry, respectively (Supplementary Table 1). Following multi-ancestry meta-analysis in the discovery phase, a total of 1,465 variants at 25 loci met a genome-wide significance threshold (*P* < 5 × 10^−8^; Supplementary Figs. 2 and 3). We replicated the known *FBN1* (ref. ^3^), *ULK4* (ref. ^4^) and *LRP1* (ref. ^4^) loci at genome-wide significance and the recently identified *TCF7L2* (ref. ^5^) locus with *P* < 5 × 10^−5^ (Supplementary Table 2). Notably, in the MVP, we found no evidence of association for three variants previously reported in an analysis of 435 thoracic aortic aneurysm cases that lacked independent replication^8^, suggesting that these rare variant associations may be false positive findings (Supplementary Table 3).

Of the 1,465 variants reaching genome-wide significance in the MVP, 1,461 were also available for independent testing in external datasets (4,459 individuals with TAAD, 512,463 individuals without TAAD across six cohorts of predominantly European ancestry) and were taken forward for replication. Following replication, 21 loci continued to exceed genome-wide significance (*P* < 5 × 10^−8^), with the four known and 17 new loci demonstrating a directionally consistent replication *P* value < 0.05 (Tables 1 and 2 and Supplementary Tables 4 and 5). The *FBN1* variant rs1818275 was the most significant association (17.4% frequency for the C allele; odds ratio (OR) = 1.35; 95% confidence interval (CI), 1.41–1.30; *P* = 2.5 × 10^−47^). All five signals that did not replicate failed to meet the pre-specified threshold (*P* < 0.05) for independent replication (Supplementary Table 6).

**Table 1:** Four known TAAD risk loci after discovery in the MVP and independent replication

Chr:pos (hg19)	rsID	EA	NEA	EAF	Overall OR	Overall 95% CI	Overall *P*	Gene/locus	Annotation
3:41,960,006	rs1716975	T	C	0.321	1.14	1.11–1.18	2.96 × 10^−15^	*ULK4*	missense_variant
10:114,773,927	rs7904519	A	G	0.519	1.09	1.06–1.12	5.71 × 10^−9^	*TCF7L2*	intron_variant
12:57,527,283	rs11172113	T	C	0.592	1.11	1.08–1.14	6.98 × 10^−16^	*LRP1*	intron_variant
15:48,883,939	rs1818275	C	T	0.174	1.35	1.30–1.41	2.54 × 10^−47^	*FBN1*	intron_variant

Abbreviations: EA, effect allele; NEA, non-effect allele; EAF, effect allele frequency; rsID, reference SNP cluster ID.

**Table 2:** Seventeen new TAAD risk loci after discovery in the MVP and independent replication

Chr:pos (hg19)	rsID	EA	NEA	EAF	Overall OR	Overall 95% CI	Overall *P*	Gene/locus*	Annotation
1:9,436,538	rs4596926	A	G	0.404	1.10	1.07–1.13	3.94 × 10^−11^	(*SPSB1*)	regulatory_region_variant
2:164,915,279	rs13002621	G	A	0.222	1.12	1.08–1.15	2.68 × 10^−12^	AC092684.1	intron_variant
2:19,729,131	rs1863777	T	C	0.361	1.14	1.11–1.17	1.67 × 10^−19^	(*OSR1*)	intergenic_variant
2:238,227,919	rs6759927	G	A	0.301	1.11	1.08–1.14	3.57 × 10^−12^	*COL6A3*	downstream_gene_variant
4:146,814,640	rs7666150	C	T	0.504	1.13	1.10–1.16	6.58 × 10^−17^	*ZNF827*	intron_variant
5:122,441,482	rs337128	C	A	0.513	1.12	1.09–1.15	2.74 × 10^−16^	*PRDM6*	intron_variant
5:95,566,562	rs55745974	A	T	0.658	1.20	1.16–1.23	1.54 × 10^−32^	*CTD-2337A12.1*	intron_variant
7:35,293,972	rs336284	A	G	0.485	1.1	1.08–1.14	6.23 × 10^−13^	*TBX20*	upstream_gene_variant
7:73,431,693	rs62465578	T	G	0.431	1.18	1.15–1.21	2.94 × 10^−31^	(*ELN*)	regulatory_region_variant
9:127,883,905	rs139650453	A	G	0.875	1.15	1.10–1.20	4.98 × 10^−10^	*SCAI*	intron_variant
10:96,061,793	rs4394764	G	A	0.810	1.15	1.11–1.19	4.52 × 10^−17^	*PLCE1*	intron_variant
11:130,271,647	rs747249	G	A	0.619	1.16	1.13–1.19	5.50 × 10^−25^	*ADAMTS8*	downstream_gene_variant
13:22,861,921	rs9316871	A	G	0.783	1.21	1.18–1.25	3.03 × 10^−32^	(AL354828.1)	regulatory_region_variant
13:50,810,171	rs2765768	G	A	0.307	1.10	1.07–1.13	4.85 × 10^−11^	*DLEU1*	intron_variant
15:71,612,514	rs1441358	T	G	0.643	1.12	1.09–1.15	4.50 × 10^−16^	*THSD4*	intron_variant
16:83,045,790	rs7500448	G	A	0.241	1.13	1.09–1.16	2.47 × 10^−13^	*CDH13*	intron_variant
17:2,097,583	rs1002135	G	T	0.398	1.11	1.09–1.14	5.10 × 10^−16^	*SMG6*	intron_variant

*Genes for variants that are outside the transcript boundary of a protein-coding gene are shown with the nearest candidate gene in parentheses (for example, (*OSR1*)).

Of the 21 TAAD risk loci, 16 were directionally consistent across European, African and Hispanic ancestries in the MVP; 13 demonstrated at least nominal significance (two-sided *P* < 0.05) in individuals of African ancestry, and six did likewise in participants of Hispanic ancestry (Supplementary Table 7). A conditional analysis with GCTA-COJO^9^ and an ancestry-matched linkage disequilibrium (LD) reference panel identified a total of five additional independent variants across the 21 replicated TAAD GWAS loci (Supplementary Table 8).

### Anatomic distribution of TAAD risk variants

**Fig. 2:**
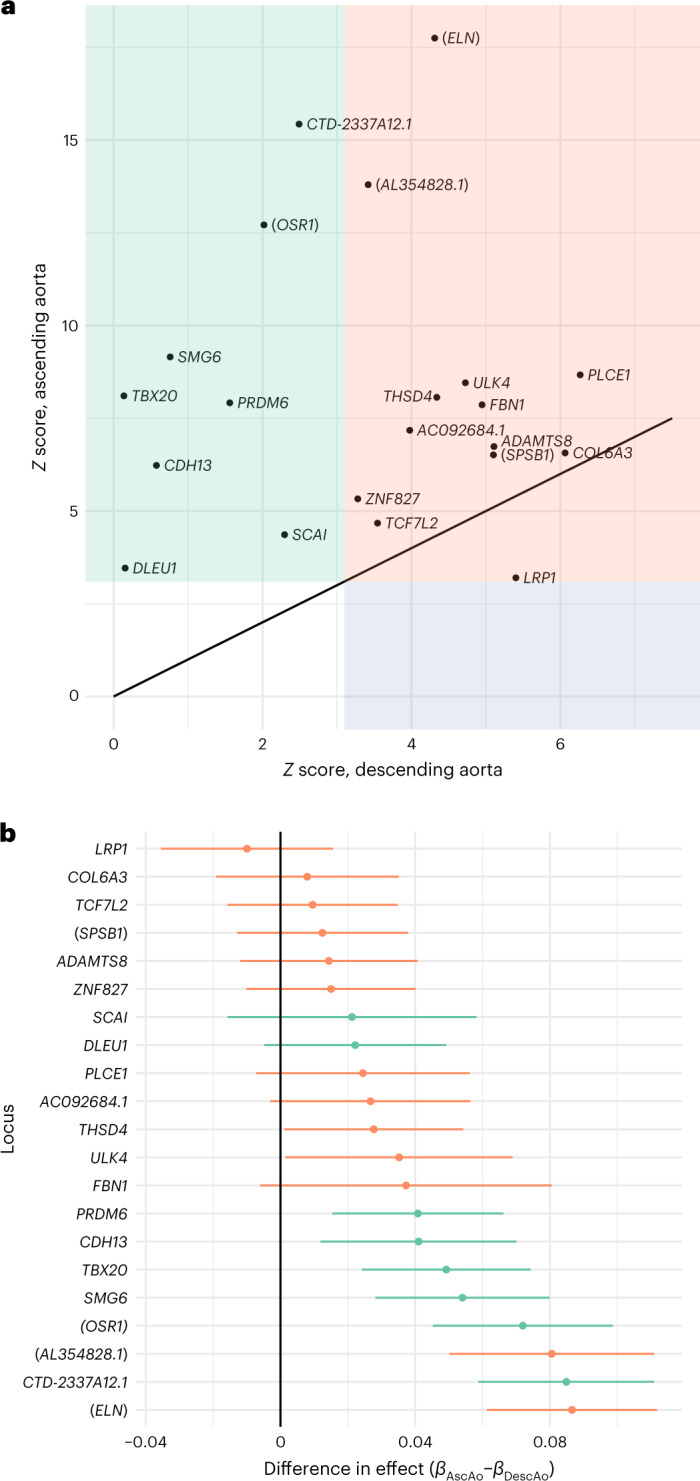
Anatomic distribution of TAAD risk variants in the thoracic aorta. **a**, Descending aortic diameter *Z* scores (*x* axis) and ascending aortic diameter *Z* scores (*y* axis) for the 21 TAAD lead risk variants in our study queried in previously published summary statistics from the UK Biobank^10^ (*n* = 39,688 individuals). **b**, Difference in effect estimates (*β*_ascending_ – *β*_descending_) and associated 95% CIs (error bars) for the 21 TAAD lead risk variants in our study queried in previously published summary statistics from the UK Biobank^10^ (*n* = 39,688 individuals). AscAo, ascending aorta; DescAo, descending aorta. Variants were declared to be significantly associated with the respective diameter if the linear mixed-model two-sided *P* value of association was <0.0012. Variants significantly associated with the diameter of the ascending aorta (green), the descending aorta (blue) or both ascending and descending aortic diameters (orange) are displayed.

We next explored whether the identified TAAD risk variants were associated with a specific anatomic distribution of aortic disease. We examined the 21 TAAD risk variants for association with ascending or descending thoracic aortic diameters in recently published UK Biobank summary statistics from Pirruccello et al. (*n* = 39,688 individuals)^10^. We found that all 21 lead variants demonstrated an association with ascending aortic diameter with Bonferroni *P* < 0.0012 (Fig. 2a,b and Supplementary Table 9). By contrast, only 13 of the 21 variants surpassed this threshold in their association with descending thoracic aortic diameter. We then examined the genome-wide significant aortic diameter loci from Pirruccello et al.^10^ within our TAAD summary statistics. Of the ascending and descending aortic diameter loci available for testing in our dataset, 49 of 81 and 12 of 46 demonstrated evidence of association with TAAD after Bonferroni correction (*P* < 0.0004), respectively (Supplementary Table 10).

### Phenotypic consequences of TAAD risk variants

Understanding the full spectrum of phenotypic consequences of a given DNA sequence variant can help identify the mechanism by which a variant or gene leads to disease. Termed PheWAS, this approach examines the association of a risk variant across a range of phenotypes^11,12^. Using data from the Integrative Epidemiology Unit (IEU) OpenGWAS project^13,14^, we performed a PheWAS of the 21 TAAD lead risk variants across a range of over 2,000 conditions, diseases and metabolites. In total, we identified 167 statistically significant (*P* < 5.0 × 10^−8^) PheWAS associations across the 21 genetic variants. In particular, several of the TAAD risk variants were associated with a range of anthropometric traits such as height and conditions including asthma and migraine with genome-wide significance (Supplementary Table 11). Notably, for seven of the 21 variants, the TAAD risk allele was associated with increased diastolic blood pressure (DBP), and three variants demonstrated an association with increased height. By contrast, for all nine variants that demonstrated an association with systolic blood pressure (SBP), the TAAD risk allele was associated with decreased SBP.

Because increased blood pressure and increased height have been reported to be risk factors for TAAD^15^, we performed a sensitivity analysis retesting the association of the seven DBP-associated variants and three height-associated variants with TAAD, accounting for DBP and height in the association model, respectively (Supplementary Methods). For DBP, while a minor decrease in the TAAD-association *P* value was observed, in each case, the *P* value remained significant at the genome-wide level, suggesting that blood pressure was not the primary mediator for the observed genetic association (Supplementary Table 12). For height, all three variants demonstrated an attenuation in association point estimate and *P* value, although this signal did not completely disappear, suggesting that some, but not all, of the association may be mediated through an increase in standing height (Supplementary Table 13).

### Causal epidemiologic risk factors for TAAD

**Fig. 3:**
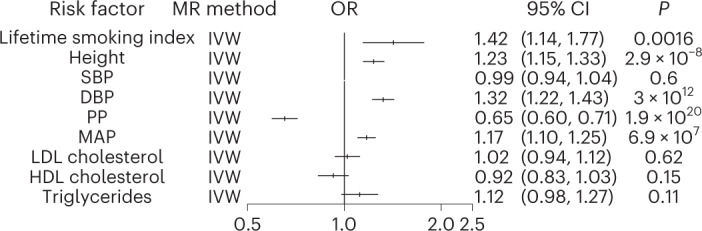
MR analyses of epidemiologic risk factors for TAAD. Logistic regression (inverse-variance-weighted; IVW) association results for multiple epidemiologic risk factor exposures with the TAAD outcome in two-sample MR analyses. The lifetime smoking TAAD OR reflects a per-genetic increase in smoking ~20 cigarettes a day for 15 years and stopping 17 years ago. The OR for height reflects a 1-s.d. genetic increase in standing height (~7.6 cm). The SBP, DBP, MAP and PP ORs correspond to the change in TAAD risk per 10-mmHg increase in the blood pressure trait. The lipid ORs reflect the change in TAAD risk per s.d. genetic increase in lipid fraction. Two-sided *P* values are displayed, and we set a two-sided *P* < 0.0055 (0.05 ÷ 9 traits) for statistical significance. Error bars represent 95% CIs of the displayed ORs.

In observational studies, smoking, hyperlipidemia, hypertension and standing height have been suggested as independent risk factors for TAAD^15,16^. We performed Mendelian randomization (MR) analyses using genetic instruments for a lifetime smoking index^17^, lipids (triglycerides, high-density lipoprotein (HDL) and low-density lipoprotein (LDL) cholesterol)^18^, blood pressure (SBP, DBP, pulse pressure (PP) and mean arterial pressure (MAP) (https://pan.ukbb.broadinstitute.org)) and height^19^ (Supplementary Table 14). Consistent with the epidemiologic literature, we observed that a 1-s.d. genetic increase in lifetime smoking index, DBP, MAP and height was associated with increased risk of TAAD (OR, 1.42 for smoking ~20 cigarettes a day for 15 years and stopping 17 years ago; 95% CI, 1.14–1.77; *P* = 0.002; OR, 1.32 per 10-mmHg increase in DBP; 95% CI, 1.22–1.43; *P* = 3.0 × 10^−12^; OR, 1.17 per 10-mmHg increase in MAP; 95% CI, 1.10–1.25; *P* = 6.9 × 10^−7^; OR, 1.23 per 7.6-cm increase in height; 95% CI, 1.15–1.33; *P* = 3.0 × 10^−8^, two-sided Bonferroni *P* < 0.0055 (Fig. 3 and Supplementary Fig. 4–6)). Conversely, a 1-s.d. genetic increase in PP was associated with a decreased risk of TAAD (OR, 0.65 per 10-mmHg increase in PP; 95% CI, 0.60–0.71; *P* = 1.9 × 10^−20^). Our results remained robust to multiple sensitivity analyses, including the weighted median^20^ as well as MR-PRESSO^21^ and MR-Egger^22^ tests for evidence of horizontal pleiotropy, although MR-PRESSO outlier-corrected results demonstrated effect estimates slightly smaller in magnitude (Supplementary Table 15a). We did not detect a significant association between a genetic 10-mmHg increase in SBP and TAAD risk or an association between any lipid fraction and TAAD.

Given the substantial overlap in risk variants associated with each blood pressure trait as well as their strong genetic correlation, we next performed an MR Bayesian model-averaging (MR-BMA) analysis, a recently developed analytic tool that applies Bayesian principles to prioritize causal risk factors among correlated exposures with shared genetic predictors (in this case, blood pressure traits)^23^. MR-BMA generates a marginal inclusion probability that prioritizes causal risk factors for disease rather than determining effect estimates for each of the blood pressure traits on TAAD risk. Genetic instruments were constructed from independent genetic variants associated with any major blood pressure-related trait (SBP, DBP, PP or MAP) at a genome-wide significance level (*P* < 5 × 10^-8^, *R*^2^ < 0.001). PP and DBP emerged as the most highly prioritized causal blood pressure traits for TAAD risk (PP marginal inclusion probability = 0.82, *P*_Nyholt_ = 2.0 × 10^−5^; DBP marginal inclusion probability = 0.68, *P*_Nyholt_ = 5.4 × 10^−3^; Supplementary Table 15b).

### Identification of candidate causal TAAD risk genes

We next sought to identify causal TAAD risk genes and variants. Prior human genetic evidence strongly suggests that *FBN1* (ref. ^24^), *ELN*^25^ and *LRP1* (refs. ^26,27^) are the causal genes at three of the identified loci, and a recent report provided evidence that *TCF7L2* is the likely causal gene acting at the locus^5^ (Supplementary Table 16). For the remaining loci, we examined the genetic literature and possible causal variants that result in a protein-altering consequence in high LD with the lead variant (*R*^2^ > 0.8). In addition, we hypothesized that TAAD risk variants may be acting by inducing expression changes locally in the proximal aortic wall and performed a fine-mapping transcriptome-wide association study (TWAS)^28^ and colocalization analyses using aortic expression quantitative trait locus (eQTL) data from the Genotype–Tissue Expression Project (GTEx)^29^. When combining the above strategies, we identified seven additional putative causal genes: *THSD4*, *COL6A3*, *CDH13*, *NOC3L*, *SCAI*, *PRDM6* and *ADAMTS8* (Supplementary Tables 16 and 17). At five of the putative causal genes, we were also able to fine map the locus to five or fewer causal variants (Supplementary Table 18).

Notably, we observed that decreased *THSD4* expression was associated with an increased risk of TAAD. The protein product of *THSD4*, ADAMTSL6, is a known microfibril-associated protein that promotes fibrillin-1 matrix assembly^30^. In a recent report, rare deleterious *THSD4* variants segregated in families with a history of thoracic aortic aneurysms, and *Thsd4*^+/−^ mice were found to have progressive thoracic aortic dilation^31^. In sum, these results suggest that common *THSD4* variants may cause TAAD through diminished gene expression in the thoracic aorta.

### Gene expression analyses reveal TAAD-relevant cell types

We next sought to identify the critical tissues and cell types for TAAD risk variants. At the genome-wide level, we first used stratified LD score regression^32^ to identify TAAD-relevant tissues and cell types. We combined publicly available expression data from GTEx^29^ and an aggregation of microarray gene expression datasets comprising 37,427 samples in human, mouse and rat^32^ (previously referred to as the ‘Franke lab dataset’) to evaluate for significant enrichment of specific tissues or cell types with genetic TAAD risk-association signals across 205 different tissues and cell types. Not surprisingly, the aorta demonstrated the strongest enrichment (*P*_enrichment_ = 6.2 × 10^−6^; Supplementary Table 19). Interestingly, we also observed an enrichment for the uterine myometrium, chondrocytes and osteoblasts (Bonferroni *P*_enrichment_ < 0.002). Human myometrium is primarily composed of smooth muscle cells, consistent with the well-recognized critical role of vascular smooth muscle cells (VSMCs) in TAAD pathogenesis. Much like fibroblasts, chondrocytes produce and maintain collagen and proteoglycans, and, in cell culture, osteoblasts are nearly indistinguishable from fibroblasts^33^, providing evidence for a key role of fibroblasts in TAAD development.

We next examined whether a putative causal TAAD risk gene set demonstrated a significant cell type enrichment. We generated a gene set of 21 putative causal TAAD risk genes: the 11 candidate causal genes identified from our GWAS as well as ten additional previously identified definitive or strong HTAAD genes (‘category A’)^6^. Of note, *FBN1* was present both in our GWAS gene set and in the HTAAD gene set. We then tested whether the 21 putative causal TAAD risk genes were enriched in specific cell types identified from publicly available single-nuclear RNA-sequencing (snRNA-seq) expression data from ascending and descending thoracic aorta^10^. Using the Fast Gene Set Enrichment software^34^, we observed that the causal TAAD gene set was significantly enriched in VSMCs (*P* = 0.0078), and a suggestive enrichment was also observed with fibroblasts (*P* = 0.02), although this was no longer significant after Bonferroni correction (*P* < 0.01 = 0.05 ÷ 5 cell types; Supplementary Table 20). These findings are consistent with our results from the stratified LD score regression analysis highlighting VSMCs and fibroblasts as causal TAAD cell types.

**Fig. 4:**
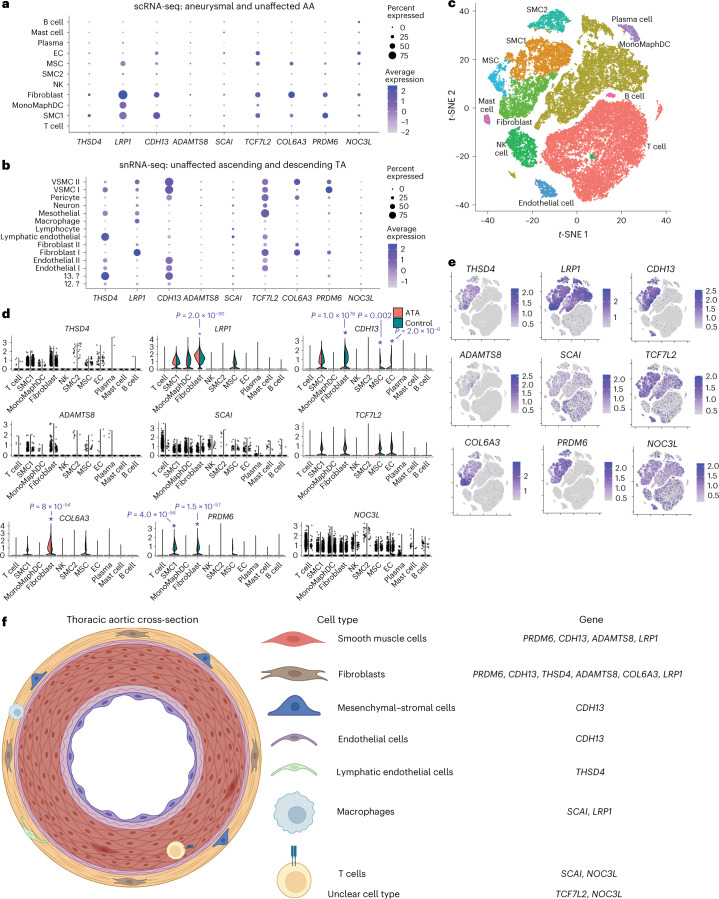
Causal TAAD cell types. Dot plots for each of the nine candidate causal genes likely affecting TAAD risk based on changes in gene expression in scRNA-seq data from aneurysmal and unaffected ascending aortas^35^ (**a**) and snRNA-seq data from unaffected ascending and descending thoracic aortas^10^ (**b**). **c**, *t*-distributed stochastic neighbor embedding (*t*-SNE) plot of cell type clusters for scRNA-seq data from aneurysmal and unaffected ascending thoracic aortas. Violin plots (**d**) and relative expression (**e**) of each of the nine candidate causal genes likely affecting TAAD risk based on changes in gene expression for each cell type. *Two-sided *P* value < 0.005 after Bonferroni correction for the maximum number of tests in each cluster (nine) when performing the Wilcoxon rank-sum test for differential expression in aneurysmal ascending thoracic aortic tissue versus unaffected tissue^35^. **f**, Prioritized cell type(s) for each of the nine candidate causal genes above depicted along a representative thoracic aortic cross-section. This figure was created with the assistance of BioRender. Abbreviations: AA, ascending aorta; TA, thoracic aorta; ATA, ascending thoracic aneurysm; scRNA-seq, scRNA-seq data; snRNA-seq, snRNA-seq data; SMC, smooth muscle cell; MonoMaphDC, monocyte–macrophage–dendritic cell; NK, natural killer cell; EC, endothelial cell; ?, unclear cell type as referenced^10^; MSC, mesothelial cell or mesenchymal–stromal cell.

Finally, we sought to identify relevant cell types for the individual putative causal TAAD risk genes leveraging single-cell RNA-sequencing (scRNA-seq) data from normal and aneurysmal ascending aortic aneurysm tissue^35^ and snRNA-seq data from normal ascending and descending thoracic aortas^10^. For the nine genes that we hypothesized promote TAAD risk through changes in aortic gene expression (‘candidate causal genes through changes in expression’ in Supplementary Table 17), we first qualitatively assessed for a prioritized cell type (1) the percentage of cells expressing the gene in a given cell type cluster and (2) the magnitude of average gene expression in each cell type (Fig. 4a–c). We then tested the differential expression of each gene among putative cell type clusters between ascending aortic aneurysm and normal aortic tissue samples using Seurat^36^ (Fig. 4d,e). Integrating this evidence, we prioritized a series of causal cell types for each gene (Fig. 4f). For example, *COL6A3* was prioritized to be acting in fibroblasts, consistent with a reported involvement in smooth muscle cell–elastin contact within the aortic wall^37^. *CDH13* (encoding cadherin 13) was prioritized to be acting in fibroblasts, mesenchymal–stromal cells and endothelial cells. Cadherin 13 signaling has been shown to be protective for endothelial cells in the setting of oxidative stress^38^, and its reported role in angiogenesis^39^ suggests that it may play a role in aortic remodeling during aneurysmal degeneration across multiple cell types. In total, we prioritized at least one candidate causal cell type for eight of the nine genes thought to be acting through changes in gene expression in the thoracic aorta.

### PRS generation for TAAD

**Fig. 5:**
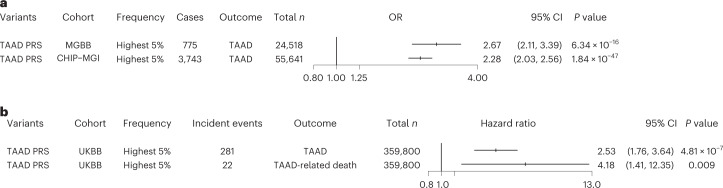
TAAD polygenic risk. **a**, Logistic regression ORs and two-sided *P* values for the association of the top 5% of the TAAD PRS with prevalent TAAD in the Mass General Brigham and CHIP–MGI biobanks. Results were combined in an inverse-variance-weighted fixed-effect meta-analysis. **b**, Hazard ratios and two-sided *P* values for the association of the top 5% of the TAAD PRS with incident TAAD and TAAD-related death in the UK Biobank. Error bars represent 95% CIs of the displayed ORs. Abbreviations: MGBB, Mass General Brigham Biobank; UKBB, UK Biobank.

Lastly, we sought to examine the contribution of polygenic inheritance on TAAD risk. We generated TAAD PRS including 1,189,073 variants from the MVP discovery GWAS summary statistics (8,626 multi-ancestry TAAD cases, 453,043 controls) and an LD panel from 1000 Genomes^40^ whole-genome-sequencing data. To increase the number of independent variants included in our score, we used the PRS-CSx software, which uses Bayesian methods to generate posterior genetic variant effect sizes under coupled continuous shrinkage priors^41^. We first validated the PRS using prevalent data from the Mass General Brigham Biobank (775 cases, 24,518 controls of European ancestry). We observed that the TAAD PRS was strongly associated with prevalent TAAD, with a 1-s.d. increase in PRS associated with a 57% increased risk of disease (OR_PRS_ = 1.57, 95% CI = 1.46–1.69, *P*_PRS_ = 4.6 × 10^−32^). Individuals with a PRS in the 95th percentile or higher were 2.67 times more likely to be diagnosed with TAAD (OR = 2.67, 95% CI = 2.11–3.39, *P*_PRS_ = 6.3 × 10^−^^16^; Fig. 5a). After this initial validation step, we then tested this PRS in two additional cohorts.

We first assessed the performance of the PRS in individuals of European ancestry in the Cardiovascular Health Improvement Project–Michigan Genomics Initiative (CHIP–MGI) cohort (3,743 cases and 51,898 controls). We again observed an effect estimate greater than 2.0 for the top 5% PRS (OR = 2.28, 95% CI = 2.03–2.56, *P*_PRS_ = 1.8 × 10^−47^; Fig. 5a). We next restricted the data to those individuals with targeted or exome sequencing available (1,842 cases and 1,887 controls) and compared the increase in area under the curve (AUC) afforded from the PRS and a set of rare TAAD risk variants that were manually curated as ‘pathogenic or likely pathogenic’ for HTAAD according to American College of Medical Genetics and Genomics best practices^42^. While the effect estimate of these pathogenic variants was substantially larger than that observed for the top 5% PRS (OR_pathogenic_ = 11.1; *P* = 1.4 × 10^−10^), we noted that, when modeling TAAD risk, the addition of the PRS improved the AUC value by a similar amount as the presence of a pathogenic TAAD risk variant (Supplementary Table 21 and Supplementary Fig. 7). In a sensitivity analysis, we noted similar results when considering rare, deleterious variants as defined by missense variants with a REVEL^43^ score >0.5 or a LOFTEE^44^ high-confidence predicted loss-of-function variant within one of the 11 HTAAD genes.

We then sought to examine whether the TAAD PRS was associated with an increase in incident TAAD and incident TAAD-related mortality. Using the UK Biobank, we first tested the PRS with all incident TAAD events and then tested the PRS with incident TAAD events listed as a primary or secondary cause of death in the electronic health record (EHR) using Cox proportional hazard models. We observed that those in the top 5% of the PRS were 2.5-fold more likely to experience an incident TAAD event during a median of 11.2 years of follow up and demonstrated more than fourfold higher risk of TAAD-related mortality (Fig. 5b).

## Discussion

In the current study, we identified 17 new TAAD loci and localized the anatomic distribution of these TAAD risk variants. We examined the phenotypic consequences of TAAD lead risk variants with PheWAS, and, through MR, we demonstrate that elevated PP and DBP, taller standing height and smoking are likely causal epidemiologic risk factors for TAAD. Leveraging bulk and scRNA-seq and snRNA-seq data, we identified causal tissues and cell types for TAAD. Lastly, we developed a genome-wide PRS for TAAD that identifies a subset of the population at substantially greater risk for TAAD.

These findings permit several conclusions. First, we provide substantial evidence that the genetic architecture of TAAD mirrors that of other complex traits. Current understanding of the pathophysiology of TAAD has largely been informed through the investigation and identification of rare deleterious variants within what are now termed HTAAD genes. These identified genetic variants, generally missense or nonsense mutations, substantially alter a gene’s protein product and subsequently disrupt critical functions in VSMC contraction, extracellular matrix stabilization or transforming growth factor (TGF)-β signaling^6^. While prior GWAS have identified four TAAD risk loci^3–5^, given the relatively rare incidence of TAAD in the population, it remained unclear whether common or rare variants were the primary driver of TAAD heritability. In the current study, we increase the number of TAAD risk loci by a factor of five and identify putative causal risk genes that likely affect disease through changes in gene expression, akin to other common complex traits. These findings offer new potential targets for therapeutic intervention as well as firmly establish TAAD as a complex trait.

Second, despite its morphologic similarities with infrarenal abdominal aortic aneurysm, our results support the notion that TAAD is a distinct disorder from the rest of the atherosclerotic cardiovascular disease spectrum. Early observational studies of TAAD often studied abdominal and thoracic aortic aneurysms together^16^, and suggested common risk factors for both diseases included hypertension, smoking and hyperlipidemia. While an association between atherosclerosis and ascending aortic aneurysms has been observed^45^, whether the two pathologic processes share underlying causal mechanisms remained unknown. Here, through causal inference methods, we provide genetic support for a causal role of smoking and hypertension on TAAD risk. However, unlike for abdominal aortic aneurysm^46,47^ and other cardiovascular diseases^48,49^, circulating lipoproteins do not appear to play a substantial role in TAAD development. The loci revealed in our genetic discovery analysis highlight the role of extracellular matrix integrity in TAAD, and the tissue and cell type enrichment analyses underscore the importance of VSMCs in TAAD risk. Prior lineage-mapping studies suggest that the embryologic origin of VSMCs in the thoracic aorta differing from that of the rest of the arterial tree may play a role in susceptibility to atherogenic conditions^50^. In light of these findings, we hypothesize that therapies focusing on restoring extracellular matrix stability rather than on atherosclerotic risk factor modification will be more likely to provide a substantial impact on TAAD prevention.

Third, our data provide additional evidence for the clinical utility of TAAD PRSs. Although recent literature focusing on PRS application has demonstrated an ability to risk stratify the population at large^51^, critics have highlighted that this research has focused on diseases in which genetic testing is seldom indicated^52^, unlikely to substantially alter clinical outcomes^53^, or that may not be justified within current healthcare cost structures^7^. However, unlike other diseases, testing for heritable causes of TAAD is already performed, typically through exome sequencing in those with familial or early-onset TAAD syndromes to identify pathogenic variants^54^. Here, we demonstrate that individuals within the upper tail of the PRS distribution are at substantially greater risk to experience TAAD or TAAD-related mortality. Furthermore, we observed similar gains in the calculated AUC statistic when adding pathogenic or likely pathogenic TAAD risk variants or PRSs (TAAD PRS) to risk-prediction models, suggesting that there is an additional benefit for PRS testing to include common variation beyond current targeted or exome-sequencing panels. In sum, our data suggest that extending current genetic panels to include testing for polygenic TAAD risk may be warranted and deserves further study.

Our study should be interpreted in the context of its limitations. First, our TAAD phenotype is based on EHR diagnosis and procedural code data and may result in misclassification of case status. However, such misclassification should, on average, reduce statistical power for discovery and bias results toward the null. Second, the VA Healthcare System population is overwhelmingly male, and our ability to detect sex-specific genetic associations in discovery was limited. Third, power to detect differential expression associations and identify causal TAAD cell types in scRNA-seq and snRNA-seq data may be limited by sequencing depth, sample size or tissue-processing techniques. Fourth, while we observe a significant MR result for height exposure and TAAD, we cannot rule out the possibility that this association is driven by the pleiotropy of height-associated variants^55^, rather than a true causal association. Similarly, when examining the scatterplots for each of the significant MR associations, it is possible that the positive results may not necessarily be attributable to a true causal effect but rather that the MR association results may reflect shared biological pathways between the risk factor and TAAD. Finally, a number of the TAAD risk loci demonstrate genome-wide significant associations with increased DBP. Our MR results support a causal role for DBP in TAAD susceptibility; however, our sensitivity analysis suggests that DBP is not the sole driver of TAAD risk at these regions of the genome. Disentangling the effects of hypertension on proximal aortic dilation and dissection, a pathologic process that alters human blood pressure homeostasis, is likely to require model systems to completely elucidate the complex mechanisms at work.

In conclusion, our data provide new mechanistic insights into TAAD risk and demonstrate that its genetic architecture is akin to that of other complex traits. We identify causal risk factors, cell types and genes that may be used to inform clinical care.

## Methods

### Study populations

We conducted a discovery genetic association analysis using DNA samples and phenotypic data from the MVP (Supplementary Fig. 1). In the MVP, individuals aged 18 to over 100 years have been recruited from 63 VA medical centers across the United States. After quality control, we identified 7,050 participants of European, 1,266 participants of African and 310 participants of Hispanic ancestry with TAAD and 453,043 controls free of clinical evidence of disease. For variants meeting genome-wide significance (*P* < 5 × 10^−8^) in the MVP, we sought replication of our findings with data from a meta-analysis of six external datasets comprising 4,459 TAAD cases and 512,463 controls (Supplementary Table 22). Additional details of the MVP and replication genetic data and quality control are available in the Supplementary Information.

### TAAD phenotype definitions

From the participants passing quality control in the MVP, individuals were defined as having TAAD based on possessing at least two of the ICD-9 or ICD-10 codes or CPT codes outlined in Supplementary Table 23 in their EHR on separate dates and possessing zero codes suggesting a possible history of bicuspid aortic valvular disease (Supplementary Table 24). Individuals were defined as not having TAAD if they had zero diagnosis or procedure codes suggesting a diagnosis of TAAD (Supplementary Table 25) and their EHR reflected two or more separate encounters in the VA Healthcare System in each of the 2 years before enrollment in the MVP. In the replication cohorts, TAAD definitions are described in Supplementary Table 22. The MVP received ethical study protocol approval by the VA Central Institutional Review Board, analysis in the UK Biobank was approved by a local institutional review board at Partners Healthcare (protocol 2013P001840), and informed consent was obtained for all participants. Additional information regarding experimental design and participants is provided in the Nature Portfolio Reporting Summary.

### Stepwise conditional analysis

We used the COJO-GCTA software^9^ to perform an approximate, stepwise conditional analysis to identify independent variants within TAAD-associated loci. We used TAAD summary statistics from the overall meta-analysis to conduct this analysis combined with an LD matrix obtained from 10,000 unrelated, ancestry-matched (86% European, 10% African, 3.5% Hispanic) individuals from the Penn Medicine BioBank. Before this conditional analysis, we aligned our summary statistics with the ancestry-matched reference panel using the DENTIST software^56^. We set a threshold *P* < 5 × 10^−8^ (genome-wide significance) to declare statistical significance.

### PheWAS of TAAD risk variants

The IEU OpenGWAS project^13,14^ is a publicly available online repository of a wide array of summary statistics from previously published GWAS and the UK Biobank. For TAAD lead variants identified in our GWAS analysis, we queried the phenotypes available in the IEU OpenGWAS project to perform a PheWAS^12^ across a range of over 2,000 conditions, diseases and metabolites. Details of the sensitivity analysis re-examining DBP and height-associated variants with TAAD accounting for blood pressure and height using individual-level data in the MVP are described in the Supplementary Methods.

### TAAD risk factor Mendelian randomization analyses

MR analyses for smoking (through a lifetime smoking index), lipid levels (triglycerides, HDL and LDL cholesterol), blood pressure (SBP, DBP, PP and MAP) and height exposure were performed with TAAD as the outcome. Given that some of the above exposure summary statistics included UK Biobank data, the TAAD outcome summary data included all of the studies in our analysis except the UK Biobank, encompassing 12,422 TAAD cases and 578,768 controls. Genetic instruments were selected as DNA sequence variants that were associated with the exposure at genome-wide significance (*P* < 5 × 10^−8^) with an *R*^2^ < 0.001. All clumping was performed using the TwoSampleMR R package^57^. Genetic instruments were constructed for the lifetime smoking index (462,690 participants)^17^, lipid levels (up to 188,577 participants)^18^, blood pressure (up to 436,845 participants; https://pan.ukbb.broadinstitute.org) and height (253,288 individuals)^19^ using publicly available summary statistics (Supplementary Table 14). Inverse-variance-weighted MR was used for the primary analysis, with weighted-median^20^ MR performed as the sensitivity analysis, allowing for up to 50% of the weight of each instrument to be drawn from invalid instruments while controlling type I error. MR-Egger^22^ analysis was performed to evaluate for horizontal pleiotropy, as was the MR-PRESSO^21^ test, which consists of three parts: (1) the global test for horizontal pleiotropy, (2) the outlier-corrected causal estimate, which corrects for the detected horizontal pleiotropy and (3) the distortion test, which tests whether the causal estimate is significantly different after outlier adjustment. Given the high genetic correlation among blood pressure traits, we then used the MR-BMA methodology^23^ to generate multivariable models for analysis to prioritize the most likely causal blood pressure traits. Further details of the MR-BMA methods are contained in the Supplementary Information.

### Causal TAAD gene and variant identification

We prioritized candidate causal genes at each of the identified TAAD risk loci by aggregating evidence from (1) prior genetic, clinical or functional studies, (2) the closest gene to the lead risk variant, (3) genes with protein-altering variants in high LD (*R*^2^ > 0.8) with the lead TAAD risk variant, (4) *cis* eQTL from the GTEx dataset in aortic tissue^29^ with association *P* < 5 × 10^−6^, (5) results from FOCUS^28^ version 0.5, a fine-mapping technique to identify causal genes in a TWAS^58^ using bulk RNA-seq data from post-mortem aortic tissue (387 individuals from GTEx) and TAAD meta-analysis (discovery and replication) summary statistics and (6) results of a colocalization analysis from our TAAD GWAS meta-analysis and eQTL data from GTEx bulk RNA-seq data in aortic tissue using the coloc R package^59^. Further methodologic details of these analyses and an analysis identifying putative causal TAAD risk variants are described in the Supplementary Information. Genes prioritized as causal (beyond *FBN1* (ref. ^24^), *ELN*^25^, *LRP1* (refs. ^26,27^) and *TCF7L2* (ref. ^5^) where prior literature has established these as the likely causal genes) were identified based on having (1) three prioritization strategies highlighting it as a likely causal gene and (2) plausible biological evidence for a role in TAAD pathogenesis based on prior genetic, clinical or functional studies.

### Stratified LD score regression analysis

As an initial enrichment analysis, we partitioned the heritability of TAAD using stratified LD score regression^32^. Stratified LD score regression leverages GWAS summary to estimate the heritability explained by each functional classification while accounting for LD structure and other annotations. For this analysis, we combined the TAAD meta-analysis summary statistics and a previously published set of 205 cell type annotations from GTEx^29^ and the previously defined ‘Franke lab dataset’ (ref. ^32^). For this analysis, we approximated the LD structure from Europeans within the 1000 Genomes^40^ reference panel and set Bonferroni-corrected *P* < 0.00024 (0.05 ÷ 205 annotations) for statistical significance.

### Enrichment analysis with human thoracic aorta snRNA-seq data

We generated a gene set of 21 putative causal TAAD risk genes: the 11 candidate causal genes identified from our GWAS as well as ten additional previously identified definitive or strong HTAAD genes (‘category A’)^6^ and overlapped our gene set with publicly available snRNA-seq data from ascending and descending thoracic aorta specimens^10^. We downloaded single-nucleus expression data and existing *t*-SNE cluster annotations for each cell from the Broad Institute Single Cell Portal (https://singlecell.broadinstitute.org/single_cell) and combined the clusters into five overarching cell categorizations: VSMCs, fibroblasts, endothelial cells, leukocytes and other cells (Supplementary Table 20). We then calculated average expression for each gene across all cells in that cell type. For each cell type, we calculated the enrichment *P* value for our list of causal TAAD risk genes using the ‘fgsea’ R package version 1.20.0, which searches for over-representation of our gene list in ranked genes for each cell type, as implemented in R 4.1. A Bonferroni two-sided *P* value < 0.01 (0.05 ÷ 5 cell types) was used to declare statistical significance.

### Cell type prioritization in scRNA-seq and snRNA-seq data

For nine genes that we hypothesized influence TAAD risk through alterations in gene expression (‘candidate causal genes through changes in expression’ in Supplementary Table 17), we prioritized causal cell types using scRNA and/or snRNA-seq data generated from thoracic aorta specimens. Previously published scRNA-seq data from control (*n* = 3) and ascending thoracic aortic aneurysm cases (*n* = 8) were reanalyzed using Seurat (version 4)^35,60^. Briefly, dimensionality reduction was previously performed using *t*-SNE, and identification of cluster-defining genes was performed using the FindAllMarkers function. Feature plots (order = TRUE, min.cutoff = ‘q1’, max.cutoff = ‘q95’, raster = TRUE, pt.size = 2.5), violin plots (split.plot = TRUE, split.by = ‘stim’), dot plots and heat maps were generated using Seurat (version 4). Within each cluster, differential gene expression based on case–control status was performed with FindAllMarkers (group.by = ‘stim’), and a Bonferroni *P* value < 0.005 (0.05 ÷ 9 maximum genes per cluster) was set for statistical significance. Previously published snRNA-seq data from the thoracic aorta were downloaded from the Broad Single Cell Portal^10^. Data from the cells were processed in R Studio with Seurat (version 4) according to the pipeline described above, with metadata of the original clustering added. Briefly, cells were filtered based on the following parameters: 250 < nFeatureRNA < 2,500, nCount_RNA > 500 and percent.mt < 0.5%. Variable features were scaled using ScaleData for percent.mt. Dimensionality reduction with UMAP was performed, and dot plots were generated with group.by = ‘Category’ corresponding to the original clusters from Pirruccello et al.^10^.

### TAAD polygenic risk score generation

A weighted PRS represents an individual’s risk of a given disease conferred by the sum of the effects of many common DNA sequence variants. A weight is assigned to each genetic variant based on its strength of association with disease risk (*β*). Individuals are then additively scored in a weighted fashion based on the number of risk alleles that they carry for each variant in the PRS.

To generate our scores, we used summary statistics from the MVP multi-ancestry discovery GWAS (8,626 TAAD cases, 453,043 controls) and an LD panel from 1000 Genomes^40^ whole-genome-sequencing data. To increase the number of independent variants included in our score, we used the PRS-CSx version 1.0 software, which uses Bayesian methods to generate posterior genetic variant effect sizes under coupled continuous shrinkage priors^41^. The latest iteration of the software allows for integration of summary statistics across multiple populations to improve polygenic predictions across ancestries. The European, African and Hispanic ancestry summary statistics were input with European, African and Admixed American reference panels from 1000 Genomes, and default software parameters were used including the use of HapMap^61^ imputed variants for PRS generation and allowing PRS-CSx to generate the global shrinkage parameter *φ* through a Bayesian approach.

We then tested our scores in three separate datasets. For initial validation, we tested the normalized PRS using prevalent data from the Mass General Brigham Biobank in 775 individuals with and 24,518 individuals without TAAD of European ancestry. Next, we assessed the performance of the PRS in an updated freeze of the CHIP–MGI cohort (3,743 cases and 51,898 controls) and subsequently compared these results (in terms of effect estimate and AUC) to the effects of a set of previously curated, pathogenic or likely pathogenic TAAD risk variants among individuals with exome-sequencing data available (a subset of 1,842 cases and 1,887 controls). In a sensitivity analysis, we also examined the effects of rare missense variants with a REVEL^43^ score >0.5 and high-confidence LOFTEE^44^ predicted loss-of-function variants in one of the 11 HTAAD genes. Lastly, we examined whether the TAAD PRS was associated with an increase in risk of incident TAAD and TAAD-related mortality within the UK Biobank among 281 participants with incident TAAD, 22 participants with TAAD-related deaths and 359,000 participants without TAAD of European ancestry.

### Statistical analysis

In our primary discovery analysis, genotyped and imputed DNA sequence variants in individuals of European, African and Hispanic ancestry were tested for association with TAAD using logistic mixed models as performed in the REGENIE version 2.0 statistical software program^62^. We included in step 1 of REGENIE (that is, prediction of individual trait values based on genetic data) variants that were directly genotyped and had a minor allele frequency >1%, <10% missingness and a Hardy–Weinberg equilibrium test *P* value > 10^−15^. The association model used in step 2 of REGENIE included as covariates age, sex and five principal components of ancestry. Next, associated statistics across MVP participants of European, African and Hispanic ancestry were meta-analyzed using an inverse-variance-weighted fixed-effect method as implemented in the METAL software program^63^. We excluded variants with a high amount of heterogeneity (*I*^2^ statistic > 75%) across the three ancestries.

For variants meeting genome-wide significance for TAAD (*P* < 5 × 10^−8^), we sought replication of our findings from a combination of six external cohorts representing 4,459 individuals with TAAD and 512,463 individuals without TAAD. Details of participant selection, quality control, phenotyping and statistical analysis are presented in Supplementary Table 22.

We defined significant new TAAD associations as those that were at least nominally significant in replication (*P* < 0.05), were directionally consistent in both cohorts and had an overall *P* < 5 × 10^−8^ (genome-wide significance) in the discovery and replication cohorts combined. New loci were defined as being more than 500,000 bp away from a known TAAD genome-wide associated lead variant. Additionally, LD information from the 1000 Genomes Project^38^ was used to determine independent variants for which the association peak extended beyond 500,000 bp. All logistic regression *P* values were two sided.

In the PheWAS analysis, DNA sequence variants were queried in the IEU OpenGWAS project^13,14^, an online resource of association statistics from previously conducted GWAS, and used a genome-wide significant *P*-value threshold (two-sided *P* < 5 × 10^−8^) to declare statistical significance.

In our MR analyses, a random-effect inverse-variance-weighted method was used as the primary analysis, with sensitivity analyses performed for the statistically significant associations as described above. We set a two-sided *P* value < 0.0055 (0.05 ÷ 9 traits) for statistical significance. For the MR-BMA analysis, the Nyholt procedure of effective tests^64^ was used to account for the strong correlation among the blood pressure traits, with a multiple-testing-adjusted *P* value = 0.05 set as the significance threshold.

In the association analysis of TAAD risk variants with ascending and descending aortic diameters, we queried previously published GWAS summary statistics from the UK Biobank in which these measurements were extracted from MRI images using a deep learning model^10^. We used a Bonferroni-corrected two-sided *P* value < 0.0012 (0.05(2 phenotypes × 21 variants)^−1^) to declare statistical significance. In the analysis examining the association of previously published aortic diameter^10^ loci with TAAD, we tested the 81 ascending and 46 descending aortic diameter autosomal loci with MAF > 0.01. We used a Bonferroni-corrected two-sided *P* value < 0.0004 (0.05(81 ascending + 46 descending loci)^−1^) to declare statistical significance.

In our PRS analysis, logistic regression models (prevalent cases) were used to estimate ORs and 95% CIs for associations of the continuous PRS (1 s.d. unit) with TAAD in the Mass General Brigham Biobank and CHIP–MGI adjusting for age, sex and five principal components. We additionally calculated the prevalence of TAAD for the 5% of individuals with the highest PRS relative to the rest of the population and generated CIs using R (version 4.1). In CHIP–MGI, we also tested the association of rare, pathogenic variants with TAAD risk using logistic regression and adjusting for age, sex and principal components. In a sensitivity analysis, we examined the TAAD risk conferred through rare missense variants with a REVEL^43^ score >0.5 and high-confidence LOFTEE^44^ predicted loss-of-function variants in one of the 11 HTAAD genes. Following analysis, an AUC statistic was generated for each of these models.

In the UK Biobank, we tested the association of the 5% of individuals with the highest TAAD PRS relative to the rest of the population with incident TAAD events and incident TAAD-related mortality using Cox proportional hazard models adjusting for age, sex and five principal components of ancestry in the white British subset of UK Biobank participants. Prevalent cases were excluded, and individuals were censored upon death, when experiencing the relevant event or at the end of follow up (a median of 11.1 years). We declared a *P* value < 0.0125 for statistical significance (0.05 ÷ 4 tests: associations for (1) the continuous PRS, (2) the top 5% PRS, (3) rare TAAD risk variants and (4) TAAD-related death). All *P* values were two-sided.

### Reporting summary

Further information on research design is available in the Nature Portfolio Reporting Summary linked to this article.

## Online content

Any methods, additional references, Nature Portfolio reporting summaries, source data, extended data, supplementary information, acknowledgements, peer review information; details of author contributions and competing interests; and statements of data and code availability are available at 10.1038/s41588-023-01420-z.

## Supplementary information

Supplementary Information Supplementary Figs. 1–7, Tables 1 and 2, Acknowledgements and Methods.

Reporting Summary

Supplementary Tables 3–25 Supplementary Tables 3–25.

## Data Availability

The full summary-level association data from the MVP TAAD discovery analysis from this study are available through dbGAP, under accession code phs001672.v10.p1 (https://www.ncbi.nlm.nih.gov/projects/gap/cgi-bin/study.cgi?study_id=phs001672.v10.p1). UK Biobank individual-level data are available for request by application (https://www.ukbiobank.ac.uk). Thoracic aortic aneurysm GWAS summary statistics from CHIP–MGI are available here: http://csg.sph.umich.edu/willer/public/TAA2021/. Individual-level Mass General Biobank data and Penn Medicine BioBank data are available at https://personalizedmedicine.partners.org/Biobank/Default.aspx and https://pmbb.med.upenn.edu/, but restrictions apply to the availability of these data, which were used under IRB approval for the current study and thus are not publicly available. Requests for the use of individual-level data from HUNT must be approved by the K.G. Jebsen Center for Genetic Epidemiology at NTNU. Applications are sent to HUNT and then discussed with the center. All scRNA-seq and snRNA-seq data were previously made publicly available at the Gene Expression Omnibus and can be accessed at GSE155468 or from the Broad Institute Single Cell Portal (https://singlecell.broadinstitute.org/single_cell).

## Code availability

Data were collected using the EasyQC package and the REGENIE version 2 software program as outlined in the Supplementary Methods. Additional software used for analysis includes the coloc R package, FOCUS–TWAS version 0.5, Eigensoft version 6, PRS-CSx version 1.0 and Seurat version 4. Clear code for analysis is available at the associated software websites, as standard analysis pipelines were used. Any code for use in the above software will be provided at reasonable request.

## References

[1] Tsai TT (2006). Long-term survival in patients presenting with type B acute aortic dissection: insights from the International Registry of Acute Aortic Dissection. Circulation.

[2] Erbel R (2014). 2014 ESC Guidelines on the diagnosis and treatment of aortic diseases: document covering acute and chronic aortic diseases of the thoracic and abdominal aorta of the adult. The Task Force for the Diagnosis and Treatment of Aortic Diseases of the European Society of Cardiology (ESC). Eur. Heart J..

[3] LeMaire SA (2011). Genome-wide association study identifies a susceptibility locus for thoracic aortic aneurysms and aortic dissections spanning *FBN1* at 15q21.1. Nat. Genet..

[4] Guo DC (2016). Genetic variants in *LRP1* and *ULK4* are associated with acute aortic dissections. Am. J. Hum. Genet..

[5] Roychowdhury T (2021). Regulatory variants in *TCF7L2* are associated with thoracic aortic aneurysm. Am. J. Hum. Genet..

[6] Renard M (2018). Clinical validity of genes for heritable thoracic aortic aneurysm and dissection. J. Am. Coll. Cardiol..

[7] Klarin, D. et al. Genetic architecture of abdominal aortic aneurysm in the Million Veteran Program. *Circulation* **142**, 1633–1646 (2020). 10.1161/CIRCULATIONAHA.120.047544 PMC7580856 32981348

[8] Ashvetiya T (2021). Identification of novel genetic susceptibility loci for thoracic and abdominal aortic aneurysms via genome-wide association study using the UK Biobank Cohort. PLoS ONE.

[9] Yang J, Lee SH, Goddard ME, Visscher PM (2011). GCTA: a tool for genome-wide complex trait analysis. Am. J. Hum. Genet..

[10] Pirruccello, J. P. et al. Deep learning enables genetic analysis of the human thoracic aorta. *Nat. Genet.* **54**, 40–51 (2021). 10.1038/s41588-021-00962-4 PMC8758523 34837083

[11] Denny JC (2010). PheWAS: demonstrating the feasibility of a phenome-wide scan to discover gene–disease associations. Bioinformatics.

[12] Denny JC (2013). Systematic comparison of phenome-wide association study of electronic medical record data and genome-wide association study data. Nat. Biotechnol..

[13] Elsworth, B. et al. The MRC IEU OpenGWAS data infrastructure. Preprint at *bioRxiv*10.1101/2020.08.10.244293 (2020).

[14] Hemani G (2018). The MR-Base platform supports systematic causal inference across the human phenome. eLife.

[15] Isselbacher EM (2005). Thoracic and abdominal aortic aneurysms. Circulation.

[16] Reed D, Reed C, Stemmermann G, Hayashi T (1992). Are aortic aneurysms caused by atherosclerosis?. Circulation.

[17] Wootton, R. E. et al. Evidence for causal effects of lifetime smoking on risk for depression and schizophrenia: a Mendelian randomisation study. *Psychol. Med*. **50**, 2435–2443 (2019). 10.1017/S0033291719002678 PMC7610182 31689377

[18] Global Lipids Genetics Consortium (2013). Discovery and refinement of loci associated with lipid levels. Nat. Genet..

[19] Wood AR (2014). Defining the role of common variation in the genomic and biological architecture of adult human height. Nat. Genet..

[20] Burgess S, Bowden J, Fall T, Ingelsson E, Thompson SG (2017). Sensitivity analyses for robust causal inference from Mendelian randomization analyses with multiple genetic variants. Epidemiology.

[21] Verbanck M, Chen CY, Neale B, Do R (2018). Detection of widespread horizontal pleiotropy in causal relationships inferred from Mendelian randomization between complex traits and diseases. Nat. Genet..

[22] Bowden J, Davey Smith G, Burgess S (2015). Mendelian randomization with invalid instruments: effect estimation and bias detection through Egger regression. Int. J. Epidemiol..

[23] Zuber V, Colijn JM, Klaver C, Burgess S (2020). Selecting likely causal risk factors from high-throughput experiments using multivariable Mendelian randomization. Nat. Commun..

[24] Marfan AB (1896). Un cas de déformation congénitale des quatres membres plus prononcée aux extrémités characterisée par lallongement des os avec un certain degré d’amincissement. Bull. Mem. Soc. Med. Hop. Paris.

[25] Szabo Z (2006). Aortic aneurysmal disease and cutis laxa caused by defects in the elastin gene. J. Med. Genet..

[26] Boucher P, Gotthardt M, Li WP, Anderson RG, Herz J (2003). LRP: role in vascular wall integrity and protection from atherosclerosis. Science.

[27] Davis FM (2015). Smooth muscle cell deletion of low-density lipoprotein receptor-related protein 1 augments angiotensin II-induced superior mesenteric arterial and ascending aortic aneurysms. Arterioscler. Thromb. Vasc. Biol..

[28] Mancuso N (2019). Probabilistic fine-mapping of transcriptome-wide association studies. Nat. Genet..

[29] GTEx Consortium. (2017). Genetic effects on gene expression across human tissues. Nature.

[30] Tsutsui K (2010). ADAMTSL-6 is a novel extracellular matrix protein that binds to fibrillin-1 and promotes fibrillin-1 fibril formation. J. Biol. Chem..

[31] Elbitar S (2021). Pathogenic variants in *THSD4*, encoding the ADAMTS-like 6 protein, predispose to inherited thoracic aortic aneurysm. Genet. Med..

[32] Finucane HK (2018). Heritability enrichment of specifically expressed genes identifies disease-relevant tissues and cell types. Nat. Genet..

[33] Ducy P, Schinke T, Karsenty G (2000). The osteoblast: a sophisticated fibroblast under central surveillance. Science.

[34] Korotkevich, G. et al. Fast gene set enrichment analysis. Preprint at *bioRxiv*10.1101/060012 (2021).

[35] Li Y (2020). Single-cell transcriptome analysis reveals dynamic cell populations and differential gene expression patterns in control and aneurysmal human aortic tissue. Circulation.

[36] Stuart T (2019). Comprehensive integration of single-cell data. Cell.

[37] Dingemans KP, Teeling P, Lagendijk JH, Becker AE (2000). Extracellular matrix of the human aortic media: an ultrastructural histochemical and immunohistochemical study of the adult aortic media. Anat. Rec..

[38] Joshi MB (2005). T-cadherin protects endothelial cells from oxidative stress-induced apoptosis. FASEB J..

[39] Philippova M (2006). Atypical GPI-anchored T-cadherin stimulates angiogenesis in vitro and in vivo. Arterioscler. Thromb. Vasc. Biol..

[40] The 1000 Genomes Project Consortium. (2015). A global reference for human genetic variation. Nature.

[41] Ruan Y (2022). Improving polygenic prediction in ancestrally diverse populations. Nat. Genet.

[42] Richards S (2015). Standards and guidelines for the interpretation of sequence variants: a joint consensus recommendation of the American College of Medical Genetics and Genomics and the Association for Molecular Pathology. Genet. Med..

[43] Ioannidis NM (2016). REVEL: an ensemble method for predicting the pathogenicity of rare missense variants. Am. J. Hum. Genet..

[44] Karczewski KJ (2020). The mutational constraint spectrum quantified from variation in 141,456 humans. Nature.

[45] Albini PT (2014). Advanced atherosclerosis is associated with increased medial degeneration in sporadic ascending aortic aneurysms. Atherosclerosis.

[46] Klarin D (2018). Genetics of blood lipids among ~300,000 multi-ethnic participants of the Million Veteran Program. Nat. Genet..

[47] Harrison SC (2018). Genetic association of lipids and lipid drug targets with abdominal aortic aneurysm: a meta-analysis. JAMA Cardiol..

[48] Do R (2013). Common variants associated with plasma triglycerides and risk for coronary artery disease. Nat. Genet..

[49] Levin MG (2021). Prioritizing the role of major lipoproteins and subfractions as risk factors for peripheral artery disease. Circulation.

[50] Majesky MW (2007). Developmental basis of vascular smooth muscle diversity. Arterioscler. Thromb. Vasc. Biol..

[51] Khera AV (2018). Genome-wide polygenic scores for common diseases identify individuals with risk equivalent to monogenic mutations. Nat. Genet..

[52] Baker E, Escott-Price V (2020). Polygenic risk scores in Alzheimer’s disease: current applications and future directions. Front. Digit. Health.

[53] Reitter-Pfoertner S (2013). The influence of thrombophilia on the long-term survival of patients with a history of venous thromboembolism. Thromb. Haemost..

[54] Wolford BN (2019). Clinical implications of identifying pathogenic variants in individuals with thoracic aortic dissection. Circ. Genom. Precis. Med..

[55] Marouli, E. et al. Rare and low-frequency coding variants alter human adult height. *Nature* **542**, 186–190 (2017). 10.1038/nature21039 PMC5302847 28146470

[56] Chen W (2021). Improved analyses of GWAS summary statistics by reducing data heterogeneity and errors. Nat. Commun..

[57] Zheng J (2017). LD Hub: a centralized database and web interface to perform LD score regression that maximizes the potential of summary level GWAS data for SNP heritability and genetic correlation analysis. Bioinformatics.

[58] Gusev A (2016). Integrative approaches for large-scale transcriptome-wide association studies. Nat. Genet..

[59] Guo H (2015). Integration of disease association and eQTL data using a Bayesian colocalisation approach highlights six candidate causal genes in immune-mediated diseases. Hum. Mol. Genet..

[60] Angueira AR (2021). Defining the lineage of thermogenic perivascular adipose tissue. Nat. Metab..

[61] Frazer KA (2007). A second generation human haplotype map of over 3.1 million SNPs. Nature.

[62] Mbatchou J (2021). Computationally efficient whole-genome regression for quantitative and binary traits. Nat. Genet..

[63] Willer CJ, Li Y, Abecasis GR (2010). METAL: fast and efficient meta-analysis of genomewide association scans. Bioinformatics.

[64] Nyholt DR (2004). A simple correction for multiple testing for single-nucleotide polymorphisms in linkage disequilibrium with each other. Am. J. Hum. Genet..

