# Supplementary material for: Genome-wide association study of thoracic aortic aneurysm and dissection in the Million Veteran Program
Source: Nat Genet. 2023 Jun 12;55(7):1106–15. doi: 10.1038/s41588-023-01420-z (PMC10335930; doi:10.1038/s41588-023-01420-z)
Supplement: Supplementary file 1 — Supplementary Figs. 1–7, Tables 1 and 2, Acknowledgements and Methods. [file 41588_2023_1420_MOESM1_ESM.pdf]

nature genetics

Article https://doi.org/10.1038/s41588-023-01420-z

# Genome-wide association study of thoracic aortic aneurysm and dissection in the Million Veteran Program

In the format provided by the authors and unedited

**Supplementary Figure 1** - Discovery thoracic aortic aneurysm and dissection genome-wide association study design

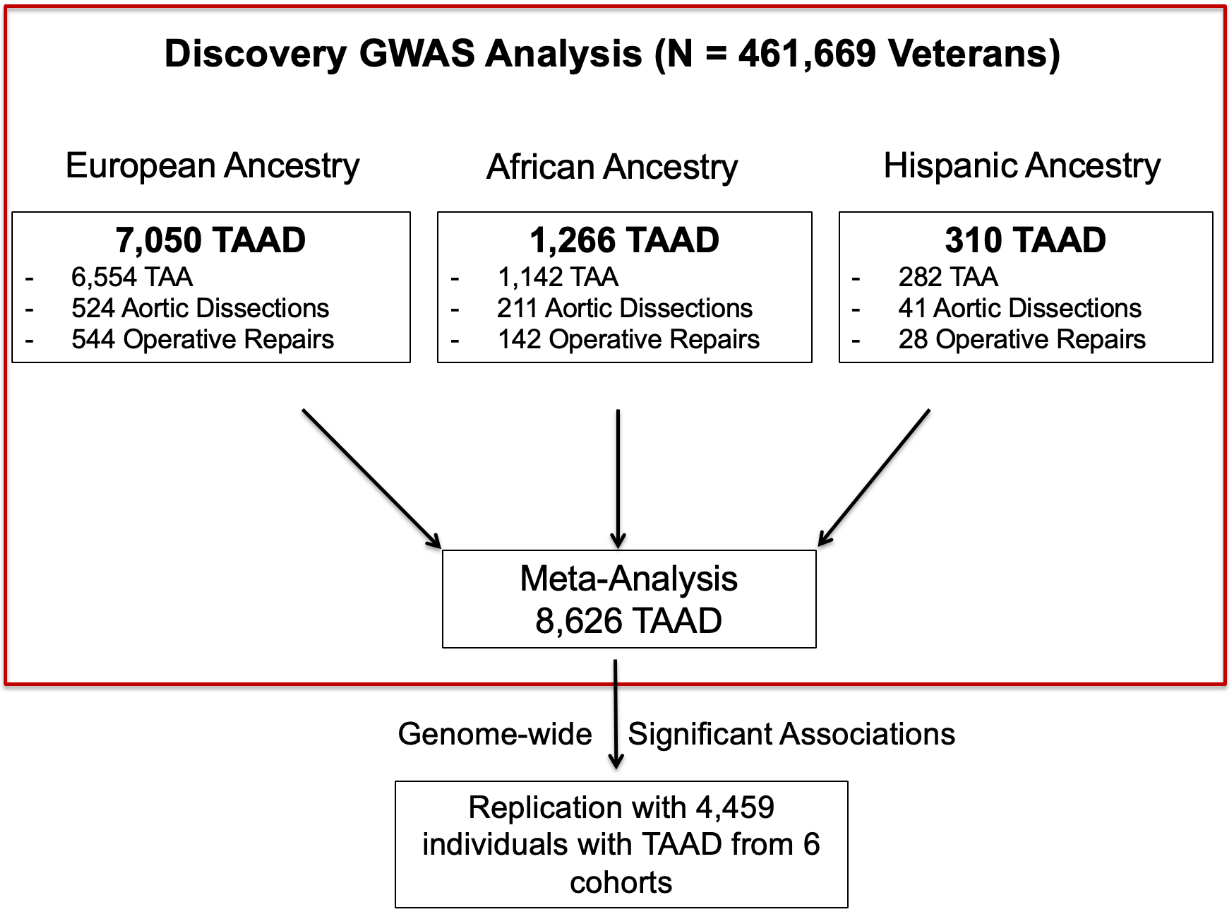


Supplementary Figure 1 **-** DNA sequence variants across 3 mutually exclusive ancestry groups in the Million Veteran Program were meta-analyzed using an inverse-variance weighted fixed effects meta-analysis in the discovery phase representing 8,626 TAAD affected individuals. Variants reaching genome-wide significance (two-sided logistic regression $P < 5x10^{-8}$) were then brought forward for independent replication in 6 external cohorts consisting of 4,459 cases and 512,463 controls.
Abbreviations: GWAS, genome-wide association study; TAAD, Thoracic Aortic Dissection and Aneurysm; TAA, Thoracic Aortic Aneurysm

**Supplementary Figure 2** - Quantile-quantile plot for the discovery multi-ancestry TAAD GWAS in MVP

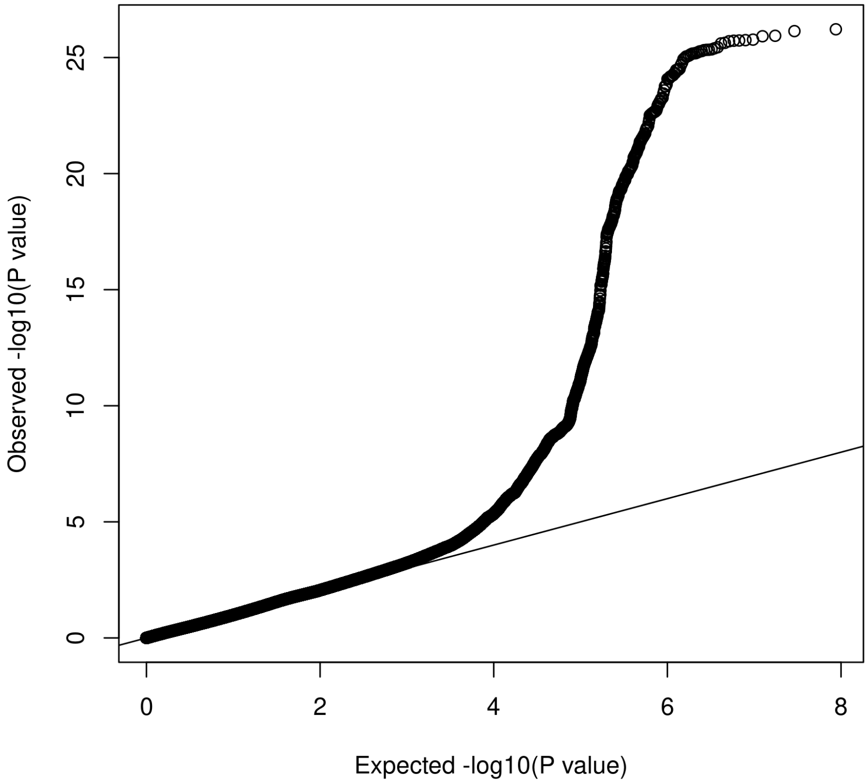


Supplementary Figure 2 - The expected logistic regression association P values versus the observed distribution of P values for TAAD association are displayed (N = 8,626 TAAD cases and 453,043 controls). Quantile-quantile plots were inspected for ancestry-specific analyses, and genomic control values were < 1.10 for each ancestry group. No systematic inflation was observed ($\lambda_{GC}$ = 1.05). All P values were two-sided. Abbreviations: TAAD, Thoracic Aortic Aneurysm and Dissection; GWAS, Genome-wide Association Study; MVP, Million Veteran Program

**Supplementary Figure 3 -** Manhattan plot for the TAAD GWAS

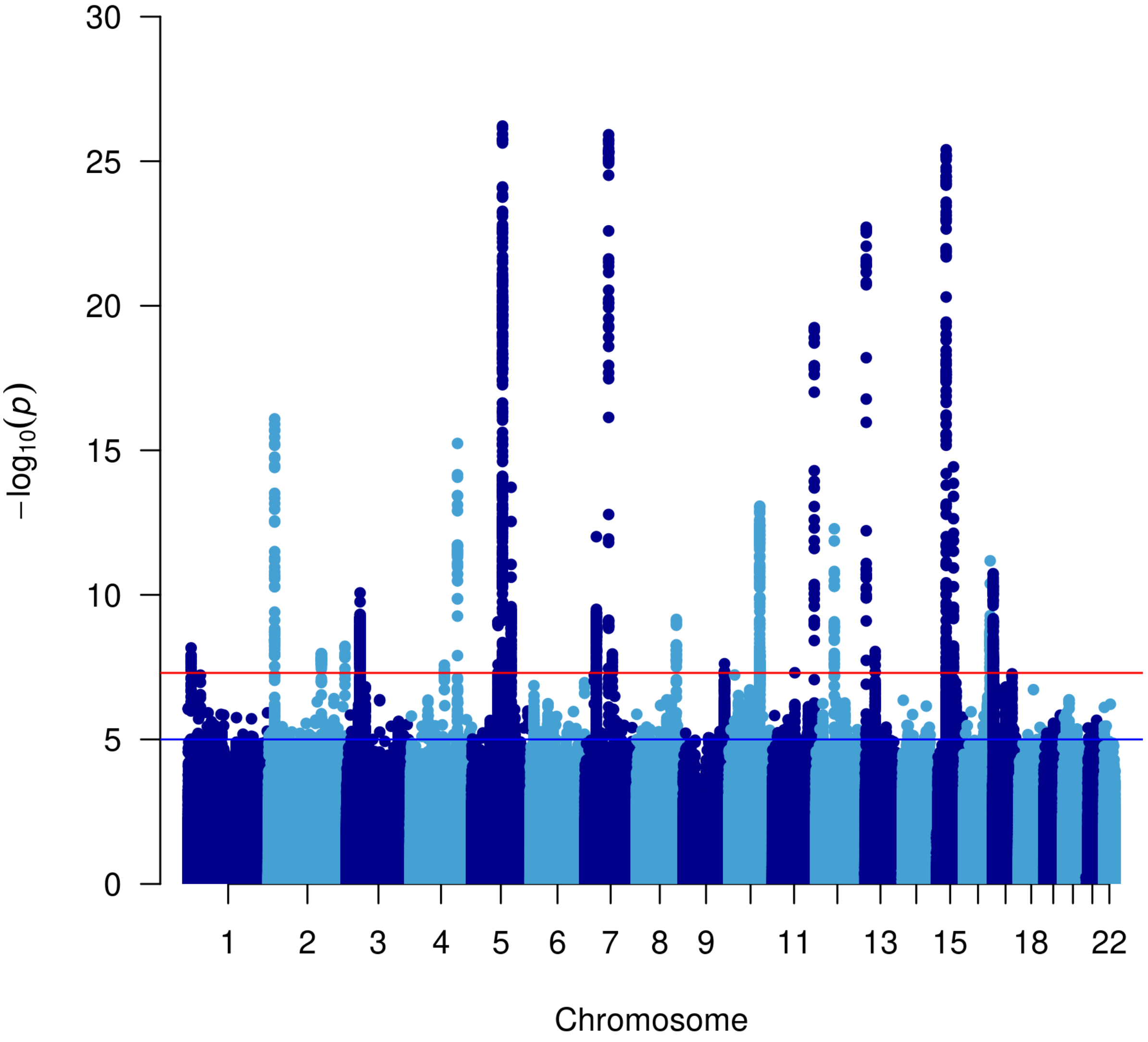


Supplementary Figure 3 - Plot of -log10(*P*) for association of genotyped and imputed variants by chromosomal position for all autosomal DNA sequence variants analyzed in the TAAD GWAS following multi-ancestry meta-analysis in MVP. Logistic regression two-sided P values are displayed.

Abbreviations: TAAD, Thoracic aortic aneurysm and dissection; GWAS, genome-wide association study; MVP, Million Veteran Program

**Supplementary Figure 4 –** Dose response plots for the blood pressure instruments tested in the causal epidemiologic risk factor Mendelian randomization analysis for TAAD

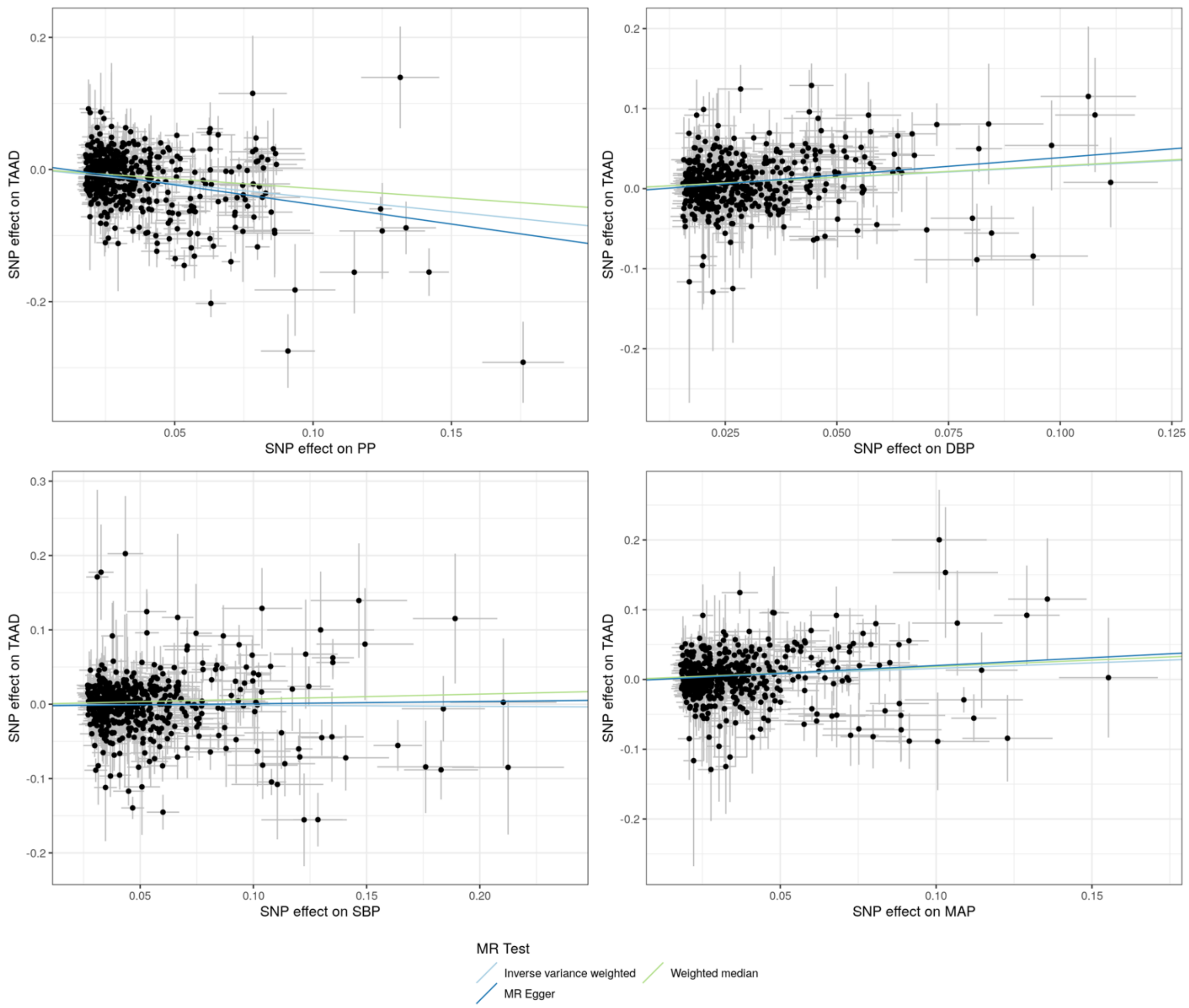


Supplementary Figure 4 – Plots of each variant's effect on the exposure (x-axis) and outcome (TAAD, Y-axis) for each of the blood pressure instruments tested in the causal epidemiologic risk factor Mendelian randomization analysis for TAAD. Lines at each point represent effect estimate confidence intervals, and regression lines for inverse variance weighted, MR Egger, and weighted median Mendelian randomization are displayed.
Abbreviations: MR, Mendelian Randomization; TAAD, Thoracic Aortic Aneurysm and Dissection; SNP, Single Nucleotide Polymorphism; DBP, Diastolic Blood Pressure; PP, Pulse Pressure; SBP, Systolic Blood Pressure; MAP, Mean Arterial Pressure

**Supplementary Figure 5 –** Dose response plots for the height instrument tested in the causal epidemiologic risk factor Mendelian randomization analysis for TAAD

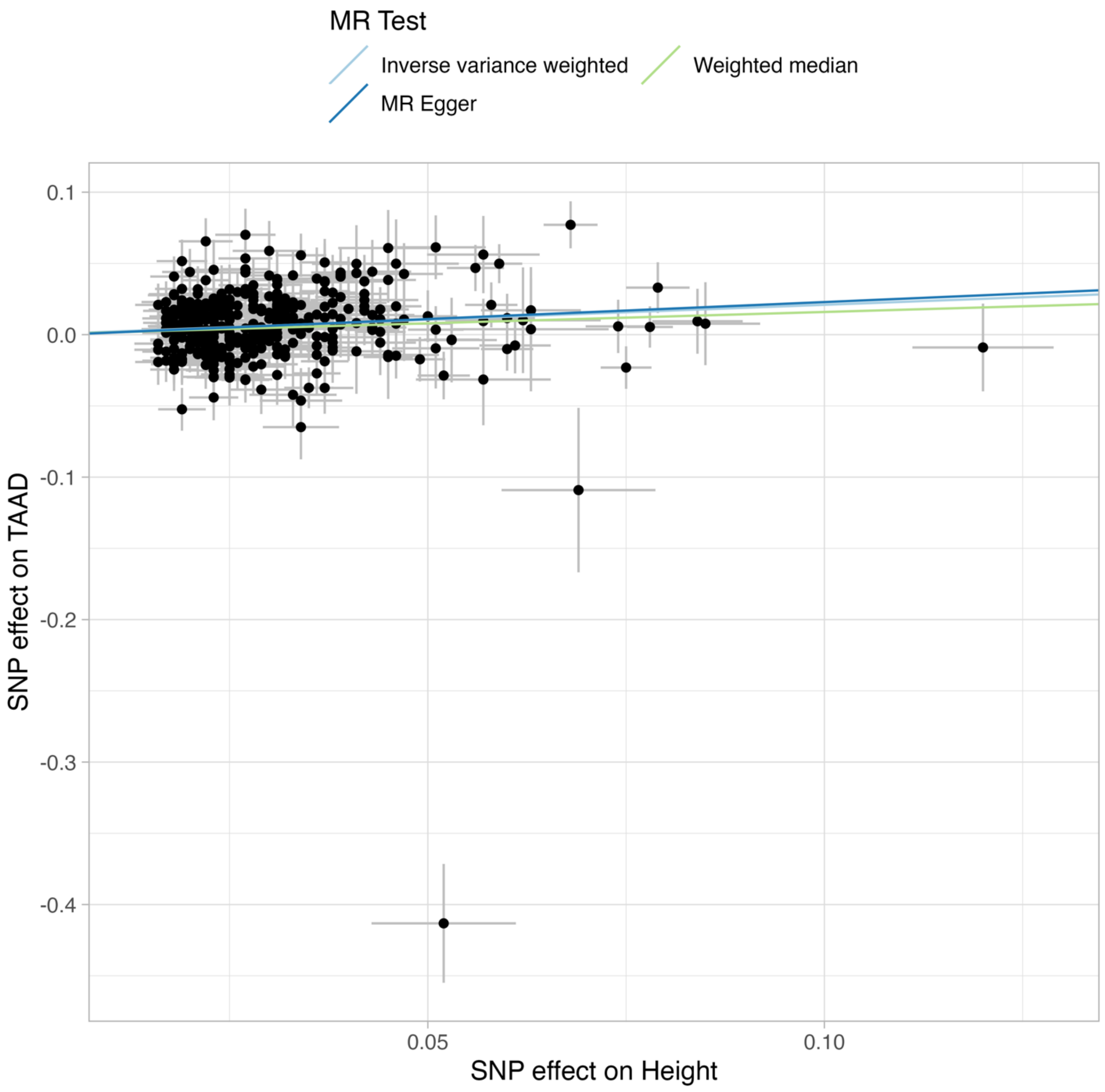


Supplementary Figure 5 – Plots of each variant's effect on the exposure (x-axis) and outcome (TAAD, Y-axis) for the height instrument tested in the causal epidemiologic risk factor Mendelian randomization analysis for TAAD. Lines at each point represent effect estimate confidence intervals, and regression lines for inverse variance weighted, MR Egger, and weighted median Mendelian randomization are displayed.

Abbreviations: MR, Mendelian Randomization; TAAD, Thoracic Aortic Aneurysm and Dissection; SNP, Single Nucleotide Polymorphism

**Supplementary Figure 6 –** Dose response plots for the lifetime smoking index instrument tested in the causal epidemiologic risk factor Mendelian randomization analysis for TAAD

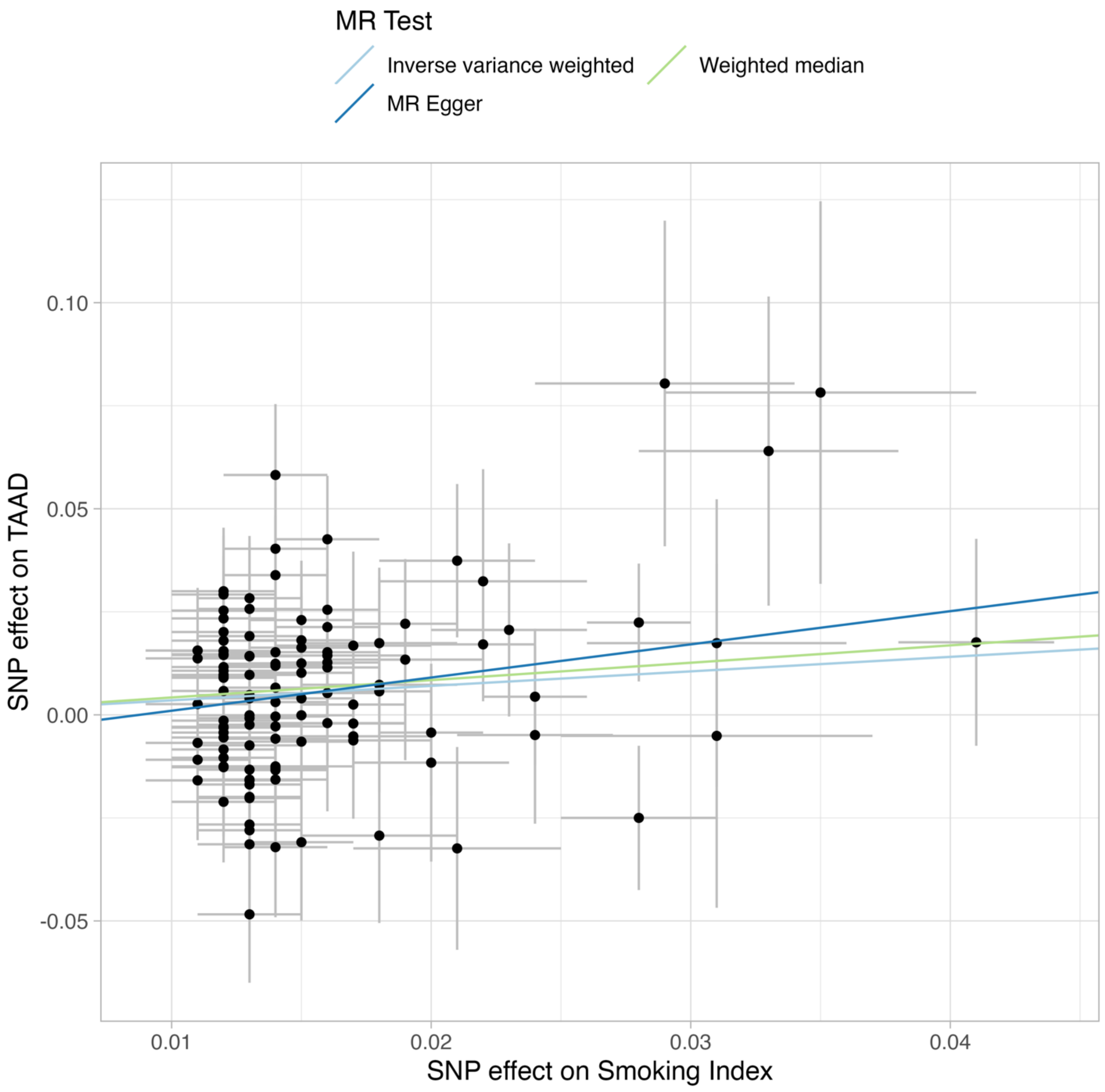


Supplementary Figure 6 – Plots of each variant's effect on the exposure (x-axis) and outcome (TAAD, Y-axis) for the lifetime smoking index instrument tested in the causal epidemiologic risk factor Mendelian randomization analysis for TAAD. Lines at each point represent effect estimate confidence intervals, and regression lines for inverse variance weighted, MR Egger, and weighted median Mendelian randomization are displayed.

Abbreviations: MR, Mendelian Randomization; TAAD, Thoracic Aortic Aneurysm and Dissection; SNP, Single Nucleotide Polymorphism

**Supplementary Figure 7 – Area under the curve for TAAD prediction for the continuous PRS per standard deviation and deleterious variants on TAAD risk in the CHIP+MGI Cohort (N = 1,842 cases and 1,887 controls with both genotyping and targeted or whole exome sequencing)**

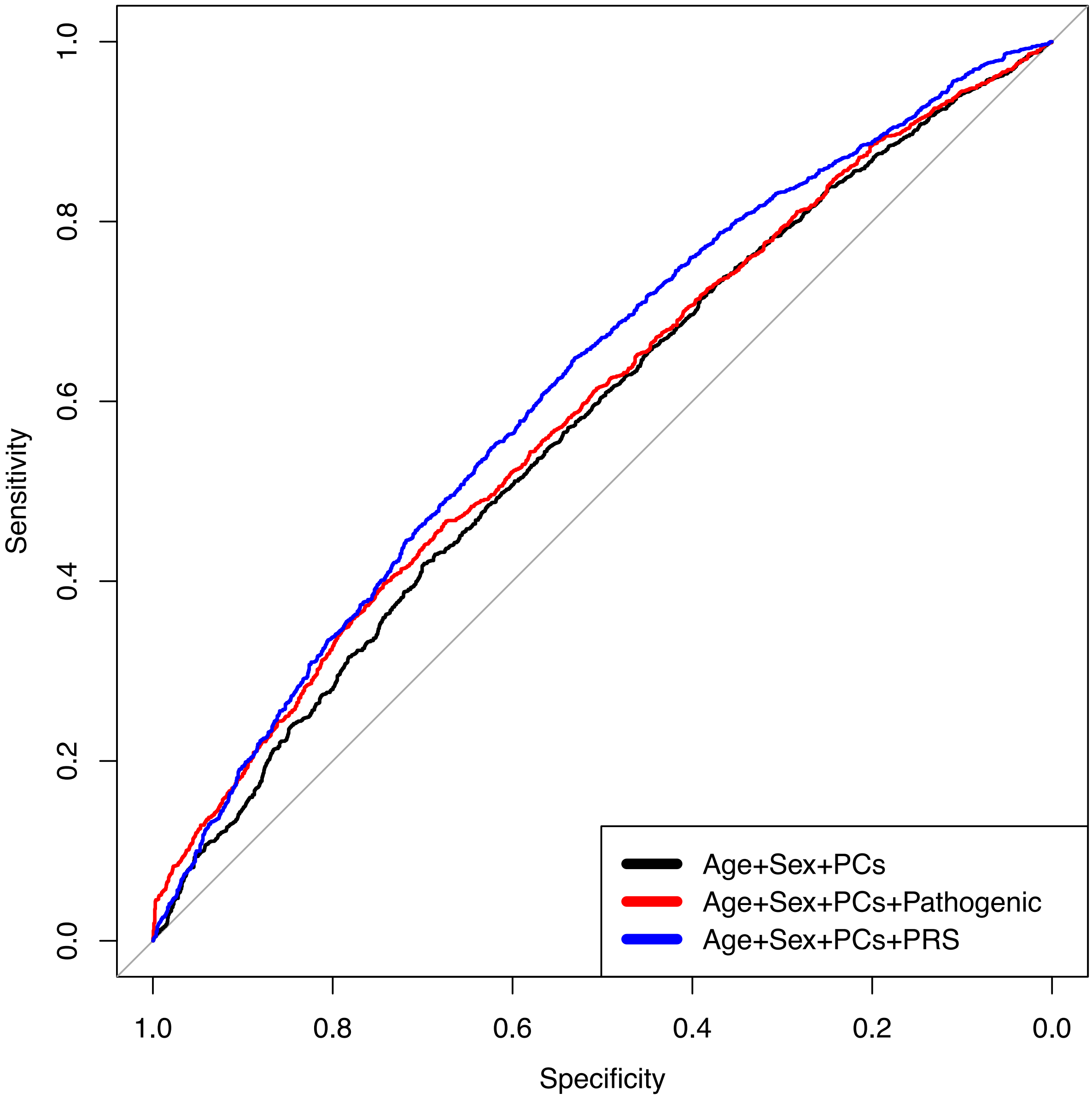


Supplementary Figure 7 – Area under the curve (AUC) performance in the CHIP-MGI cohort of 1) a "baseline model" of age, sex, and principal components, 2) the baseline model plus a set of rare TAAD risk variants that were manually curated as "pathogenic or likely pathogenic" for Heritable TAAD according to ACMG best practices[1], and 3) the baseline model plus PRS per standard deviation increase constructed based on the MVP discovery TAAD summary statistics (7,050 European, 1,266 African, and 310 Hispanic ancestry individuals with TAAD and 453,043 participants without TAAD).
Abbreviations: PCs, Principal Components; PRS, Polygenic Risk Score

**Supplementary Table 1 -** Demographic and clinical characteristics for Veterans in the MVP discovery TAAD GWAS analysis

| | **European** | | **African** | | **Hispanic** | |
|---|---|---|---|---|---|---|
| | TAAD Cases | TAAD Controls | TAAD Cases | TAAD Controls | TAAD Cases | TAAD Controls |
| Veterans, N | 7,050 | 330,610 | 1,266 | 88,107 | 310 | 34,326 |
| Age ± SD, years | 69.3 ± 8.8 | 64.7 ± 13.1 | 64.9 ± 9.4 | 58.5 ± 11.8 | 66.4 ± 9.6 | 56.8 ± 14.9 |
| Male, n (%) | 6,930 (98.3%) | 305,780 (92.5%) | 1,235 (97.6%) | 75,893 (86.1%) | 298 (96.1%) | 31,222 (91.0%) |
| Statin Therapy Prescription, n (%) | 6,094 (86.4%) | 244,330 (73.9%) | 1,117 (88.2%) | 62,434 (70.9%) | 253 (84.8%) | 22,841 (66.5%) |
| Diabetes, n (%) | 2,538 (36.0%) | 124,502 (37.8%) | 587 (46.4%) | 40,173 (45.6%) | 128 (41.3%) | 15,008 (43.7%) |
| Former Smoker, n (%) | 4,786 (67.9%) | 213,498 (64.6%) | 809 (63.9%) | 47,816 (54.3%) | 231 (74.5%) | 21,847 (63.6%) |
| Current Smoker, n (%) | 1,414 (20.0%) | 63,553 (19.2%) | 334 (26.3%) | 22,279 (25.3%) | 46 (14.8%) | 5,620 (16.4%) |
| Variants Passing Quality Control | 25,477,799 | | 40,262,025 | | 34,883,132 | |

Abbreviations: SD, Standard Deviation; TAAD, Thoracic Aortic Dissection and Aneurysm; GWAS, Genome-wide Association Study

**Supplementary Table 2 –** Logistic regression odds ratios and two-sided P values (likelihood ratio test) for four lead autosomal, genome-wide associated TAAD variants previously reported in the literature and the lead variant and two-sided P values in MVP.

| Published Lead Variant | Chr | Locus | Published Beta | Published P Value | MVP Lead Variant* | MVP Beta | $R^{2†}$ | MVP P Value | Ref |
|---|---|---|---|---|---|---|---|---|---|
| rs2118181 | 15 | *FBN1* | 0.59 | $5.9x10^{-12}$ | rs1818275 | 0.27 | 0.87 | $4.0x10^{-26}$ | 2 |
| rs11172113 | 12 | *LRP1* | 0.20 | $2.7x10^{-8}$ | rs11172113 | 0.11 | NA | $5.2x10^{-13}$ | 3 |
| rs2272007 | 3 | *ULK4* | 0.30 | $1.2x10^{-9}$ | rs12330747 | 0.12 | 0.89 | $8.5x10^{-11}$ | 3 |
| rs4073288‡ | 10 | *TCF7L2* | 0.25 | $5.2x10^{-9}$ | rs765777 | 0.07 | 0.003 | $3.7x10^{-5}$ | 4 |

* Defined as the lead variant in MVP within a 500kB window around previously published lead variant
† $R^2$ value based on European 1000 Genomes[5] reference panel
‡ Variant rs4073288 demonstrates a P = 0.003 in MVP multi-ancestry discovery GWAS
Abbreviations: Chr, chromosome; OR, Odds Ratio; Ref, Reference; GWAS, Genome-wide Association Study; MVP, Million Veteran Program

## Supplementary Methods

*Quality Control Analysis*

In MVP, we excluded: duplicate samples, samples with more heterozygosity than expected, an excess (>2.5%) of missing genotype calls, or discordance between genetically inferred sex and phenotypic gender. In addition, one individual from each pair of related individuals (kinship > 0.0884 as measured by the KING[6] software) were removed. Veterans were then divided into three mutually exclusive ethnic groups based on DNA extracted from whole blood was genotyped in MVP using a customized Affymetrix Axiom biobank array, the MVP 1.0 Genotyping Array. Veterans were divided into three mutually exclusive populations using the HARE algorithm[7]: 1) non-Hispanics of European ancestry, 2) non-Hispanics of African ancestry, and 3) Hispanics. Prior to imputation, variants that were poorly called or that deviated from Hardy-Weinberg equilibrium or their expected allele frequency based on reference data from the 1000 Genomes Project[5] were excluded. After pre-phasing using SHAPE-IT4[8], genotypes from the African Genome Resources reference panel were imputed into Million Veteran Program (MVP) participants via Minimac4 software[9]. Ethnicity-specific principal component analysis was performed using the EIGENSOFT v6 software[10].

Following imputation, variant level quality control was performed using the EasyQC R package[11] (www.R-project.org), and exclusion metrics included: imputation quality <0.3, minor allele frequency (MAF) < 0.005, call rate < 97.5% for common variants (MAF > 1%), and call rate < 99% for rare variants (MAF < 1%). Variants were also excluded if they deviated > 10% from their expected allele frequency based on reference data from the 1000 Genomes Project[5]. Quality control metrics for replication cohorts are depicted in **Supplementary Table 22**.

*Analysis adjusting the TAAD association for DBP and standing height*

To evaluate the conditional effects of the DBP and height associated variants on TAAD after accounting for these risk factors, we re-tested their association with TAAD after including DBP or standing height as a covariate in the association model in the European, African, and Hispanic ancestry participants in MVP, stratified by ancestry. Association analysis was performed using the REGENIE v2.0 statistical software program[12] adjusting for age, sex, and 5 principal components of ancestry as in the primary model.

*Fine-mapped Transcriptome-wide Association Study (TWAS)*

We performed a fine-mapped TWAS[13] using the FOCUS v0.6 software[14]. This technique leverages expression weights from bulk RNA-seq data from post-mortem aorta tissue from the Genotype-Tissue Expression project[15] (GTEx V6), and combined TAAD meta-analysis summary statistics yielding candidate causal genes from the GWAS results under the assumption that the causal mechanism of the tested genes involves changes in *cis*-expression. Briefly, this approach integrates information from expression reference panels (variant–expression correlation), GWAS summary statistics (variant–trait correlation), and linkage disequilibrium (LD) reference panels (variant–variant correlation) to assess the association between the *cis*-genetic component of expression and phenotype[13]. The results are then fine-mapped leveraging genetic variant fine-mapping approaches[16] into a 90%-credible set. We selected genes with a marginal posterior inclusion probability (PIP) > 0.2 as evidence identifying a candidate causal gene. In a sensitivity analysis, we restricted the input TAAD summary statistics to individuals of European ancestry, and our results were observed to be unchanged.

*Colocalization Analysis*

We identified genome-wide significant signals in our TAAD GWAS meta-analysis that successfully replicated and performed colocalization analysis using the *coloc*[17] tool. To identify putative causal genes and variants, we formally tested for shared association signals between expression quantitative trait loci (eQTLs) in GTEx bulk RNA-seq data from post-mortem aortic tissue (387 individuals from V8) and our TAAD meta-analysis summary statistics. We performed colocalization within a 1 mB window (+/- 500 kB) around the lead TAAD risk variant, and defined a conditional probability of colocalization (PP4) of greater than or equal to 0.9 as significant. We then additionally report loci/variants in situations when the resultant 99% credible set of causal variants identified by the *coloc* software identified 5 or fewer causal variants.

*MR-BMA Analysis*

Genetic associations between BP traits (exposure) and the TAAD outcome were tested initially using inverse-variance weighted MR for a single BP exposure, and then using the MR-BMA methodology for multivariable models[18]. MR-BMA is an extension of multivariable MR utilizing a Bayesian variable selection method in an effort to identify likely causal risk factors among correlated exposures. In the primary analysis, the instrumental variables consisted of independent genetic variants ($r^2 < 0.001$ based on 1000 Genomes[5] European ancestry Reference Panel) associated with any BP trait at genome-wide significance in the Pan UKBB analysis[19] of up to 436,845 European-ancestry participants. Genetic associations with BP traits (SBP, DBP, PP, MAP) were used as exposures. The subsequent MR-BMA analysis was completed using TAAD GWAS summary statistics from the current study, with the exception of removing UK Biobank data from the TAAD GWAS summary statistics to minimize sample overlap. Variable selection was based on marginal inclusion probabilities for which an empirical permutation procedure was used to derive P values. The Nyholt procedure of effective tests was used to account for the strong correlation among the BP traits with a multiple testing–adjusted P value of P=0.05 set as the significance threshold[20].

MR-BMA performs variable selection by evaluating models with all possible combinations of BP-related traits as exposures and computing the posterior probability that the model contains the true causal risk factors. Unlike other univariate or multivariable MR methods, MR-BMA aims to identify true causal risk factors among correlated traits, rather than estimate the magnitude of effect. The marginal inclusion probability (level of evidential support for each exposure) is derived from the sum of all posterior probabilities of the models where the specific exposure was included. We removed influential variants based on the Cook's distance and outliers based on the q-statistic as previously recommended[21]. An empirical permutation procedure was performed to calculate p-values. Briefly, the expected marginal inclusion probability distribution for each risk factor under the null hypothesis was generated by performing 1,000 permutations of the MR-BMA analysis, holding the SNP-risk factor associations constant and randomly permuting the SNP-outcome associations. The observed marginal inclusion probabilities for each risk factor were then compared to the expected distribution under the null, with p-values computed by pj = (rj +1)/(nperm + 1), where rj represents the rank of the observed marginal inclusion probability of a given risk factor (j) across all permutations (nperm = 1000). Adjustment for multiple testing was done using the Nyholt correction for correlated traits[20].

*Replication Cohort Descriptions*
For each cohort, genotyping platform, quality control metrics, phenotype definitions, and participant counts/ancestry are provided in **Supplementary Table 22**.

CHIP-MGI
The Cardiovascular Health Improvement Project (CHIP) is a cohort of individuals treated at Michigan Medicine with linked genotype, EHR, and family history data. The Michigan Genomics Initiative (MGI) is a hospital-based cohort with linked genotype and EHR data from participants recruited during pre-surgical encounters at Michigan Medicine.

Penn Medicine Biobank
Penn Medicine Biobank (PMBB) recruits patients from throughout the University of Pennsylvania Health System for genomic and precision medicine research. Participants actively consent to allow the linkage of biospecimens to their longitudinal EHR. Currently, >60 000 participants are enrolled in the PMBB. A further subset of ~23,000 subjects with imputed genotype data was used in this analysis.

UK Biobank
The UKB is a population-based cohort of approximately 500,000 participants recruited from 2006-2010 with existing genomic and longitudinal phenotypic data and median 10-year follow-up[22]. Baseline assessments were conducted at 22 assessment centres across the UK with sample collections including blood-derived DNA. Use of the data was facilitated through UK Biobank Application 7089.

MassGeneral Brigham Biobank
The MGBB contains genotypic and clinical data from >105,000 patients who consented to broad-based research across 7 regional hospitals and median 3-year follow-up[23]. Baseline phenotypes were ascertained from the electronic medical record and surveys.

HUNT
The Nord-Trøndelag Health Study (HUNT) is a population-based health survey conducted in the county of Nord-Trøndelag, Norway, since 1984. Individuals were included at three different time points during approximately 20 years of follow up.

University of Texas Health Science Center at Houston
Data contributed from this study comprised a GWAS of 765 individuals with sporadic ascending aortic aneurysms or classic aortic dissection of the ascending or descending thoracic aorta (Stanford types A and B, respectively) who presented for treatment at the Texas Medical Center. The diagnosis of TAAD was confirmed by cross-sectional imaging in all subjects and by direct inspection during surgical repair in most subjects. Controls were individuals free of disease, as described in LeMaire et al[2].

**Supplementary Acknowledgements**

This publication does not represent the views of the Department of Veteran Affairs or the United States Government. Use of the data was facilitated through UK Biobank Application 7089. The authors acknowledge the Michigan Genomics Initiative participants, Precision Health at the University of Michigan, the University of Michigan Medical School Central Biorepository, and the University of Michigan Advanced Genomics Core for providing data and specimen storage, management, processing, and distribution services, and the Center for Statistical Genetics in the Department of Biostatistics at the School of Public Health for genotype data curation, imputation, and management in support of the research reported in this publication/grant application/presentation. We acknowledge the Penn Medicine BioBank (PMBB) for providing data and thank the patient-participants of Penn Medicine who consented to participate in this research program. We would also like to thank the Penn Medicine BioBank team and Regeneron Genetics Center for providing genetic variant data for analysis. The PMBB is approved under IRB protocol# 813913 and supported by Perelman School of Medicine at University of Pennsylvania, a gift from the Smilow family, and the National Center for Advancing Translational Sciences of the National Institutes of Health under CTSA award number UL1TR001878.

**Regeneron Genetics Center Banner Author List and Contribution Statements**

**RGC Management and Leadership Team**
Goncalo Abecasis, D.Phil. , Aris Baras, M.D. , Michael Cantor, M.D. , Giovanni Coppola, M.D. , Andrew Deubler , Aris Economides, Ph.D. , Katia Karalis, Ph.D. , Luca A. Lotta, M.D., Ph.D. , John D. Overton, Ph.D. , Jeffrey G. Reid, Ph.D. , Katherine Siminovitch, M.D. , Alan Shuldiner, M.D.

**Sequencing and Lab Operations**
Christina Beechert , Caitlin Forsythe, M.S. , Erin D. Fuller , Zhenhua Gu, M.S. , Michael Lattari , Alexander Lopez, M.S., John D. Overton, Ph.D. , Maria Sotiropoulos Padilla, M.S. , Manasi Pradhan, M.S. , Kia Manoochehri, B.S. , Thomas D. Schleicher, M.S. , Louis Widom , Sarah E. Wolf, M.S. , Ricardo H. Ulloa, B.S.

**Clinical Informatics**
Amelia Averitt, Ph.D. , Nilanjana Banerjee, Ph.D. , Michael Cantor, M.D. , Dadong Li, Ph.D. , Sameer Malhotra, M.D. , Deepika Sharma, MHI , Jeffrey Staples , Ph.D.

**Genome Informatics**
Xiaodong Bai, Ph.D. , Suganthi Balasubramanian, Ph.D. , Suying Bao, Ph.D. , Boris Boutkov, Ph.D. , Siying Chen, Ph.D. , Gisu Eom, B.S. , Lukas Habegger, Ph.D. , Alicia Hawes, B.S. , Shareef Khalid , Olga Krasheninina, M.S. , Rouel Lanche, B.S. , Adam J. Mansfield, B.A. , Evan K. Maxwell, Ph.D. , George Mitra, B.A. , Mona Nafde, M.S. , Sean O'Keeffe, Ph.D. , Max Orelus, B.B.A. , Razvan Panea, Ph.D. , Tommy Polanco, B.A. , Ayesha Rasool, M.S. , Jeffrey G. Reid, Ph.D. , William Salerno, Ph.D. , Jeffrey C. Staples, Ph.D. , Kathie Sun, Ph.D. , Jiwen Xin, Ph.D.

**Analytical Genomics and Data Science**
Goncalo Abecasis, D.Phil. , Joshua Backman, Ph.D. , Amy Damask, Ph.D. , Lee Dobbyn, Ph.D. , Manuel Allen Revez Ferreira, Ph.D. , Arkopravo Ghosh, M.S. , Christopher Gillies, Ph.D. , Lauren Gurski, B.S. , Eric Jorgenson, Ph.D. , Hyun Min Kang, Ph.D. , Michael Kessler, Ph.D. , Jack Kosmicki, Ph.D. , Alexander Li , Ph.D. , Nan Lin, Ph.D. , Daren Liu, M.S. , Adam Locke, Ph.D. , Jonathan Marchini, Ph.D. , Anthony Marcketta, M.S. , Joelle Mbatchou, Ph.D. , Arden Moscati, Ph.D. , Charles Paulding, Ph.D. , Carlo Sidore, Ph.D. , Eli Stahl, Ph.D. , Kyoko Watanabe, Ph.D. , Bin Ye, Ph.D. , Blair Zhang, Ph.D. , Andrey Ziyatdinov, Ph.D.

**Therapeutic Genetics**
Luca A. Lotta, M.D., Ph.D., George Hindy, M.D., Ph.D., Niek Verweij, Ph.D., Jonas B. Nielsen, M.D., Ph.D., Tanima De, Ph.D.

**Research Program Management & Strategic Initiatives**
Marcus B. Jones, Ph.D. , Michelle G. LeBlanc, Ph.D., Jason Mighty, Ph.D. , Lyndon J. Mitnaul, Ph.D.

## VA Million Veteran Program

**MVP Executive Committee**

- Co-Chair: J. Michael Gaziano, M.D., M.P.H.
- Co-Chair: Rachel Ramoni, D.M.D., Sc.D.
- Jean Beckham, Ph.D.
- Jim Breeling, M.D. (ex-officio)
- Kyong-Mi Chang, M.D.
- Grant Huang, Ph.D. (ex-officio)
- Sumitra Muralidhar, Ph.D.
- Christopher J. O'Donnell, M.D., M.P.H.
- JP Casas Romero, M.D., Ph.D., Ex-Officio
- Philip S. Tsao, Ph.D.

**MVP Program Office**

- Sumitra Muralidhar, Ph.D.
- Jennifer Moser, Ph.D.

**MVP Recruitment/Enrollment**

- Recruitment/Enrollment Director/Deputy Director, Boston – Stacey B. Whitbourne, Ph.D.; Jessica V. Brewer, M.P.H.
- MVP Coordinating Centers
  - Clinical Epidemiology Research Center (CERC), West Haven – John Concato, M.D., M.P.H.
  - Cooperative Studies Program Clinical Research Pharmacy Coordinating Center, Albuquerque - Stuart Warren, J.D., Pharm D.; Dean P. Argyres, M.S.
  - Genomics Coordinating Center, Palo Alto – Philip S. Tsao, Ph.D.
  - Massachusetts Veterans Epidemiology Research Information Center (MAVERIC), Boston - J. Michael Gaziano, M.D., M.P.H.
  - MVP Information Center, Canandaigua – Brady Stephens, M.S.
- Core Biorepository, Boston – Mary T. Brophy M.D., M.P.H.; Donald E. Humphries, Ph.D.
- MVP Informatics, Boston – Nhan Do, M.D.; Shahpoor Shayan
- Data Operations/Analytics, Boston – Xuan-Mai T. Nguyen, Ph.D.

**MVP Science**

- Genomics - Christopher J. O'Donnell, M.D., M.P.H.; Saiju Pyarajan Ph.D.; Philip S. Tsao, Ph.D.
- Phenomics - Kelly Cho, M.P.H, Ph.D.
- Data and Computational Sciences – Saiju Pyarajan, Ph.D.
- Statistical Genetics – Elizabeth Hauser, Ph.D.; Yan Sun, Ph.D.; Hongyu Zhao, Ph.D.

**MVP Local Site Investigators**

- Atlanta VA Medical Center (Peter Wilson)
  1670 Clairmont Rd, Decatur, GA 30033

- Bay Pines VA Healthcare System (Rachel McArdle)
  10,000 Bay Pines Blvd Bay Pines FL 33744
- Birmingham VA Medical Center (Louis Dellitalia)
  700 S. 19th Street Birmingham AL 35233
- Cincinnati VA Medical Center (John Harley)
  3200 Vine Street, Cincinnati, OH 45220
- Clement J. Zablocki VA Medical Center (Jeffrey Whittle)
  5000 West National Avenue, Milwaukee, WI 53295
- Durham VA Medical Center (Jean Beckham)
  508 Fulton Street   Durham, NC 27705
- Edith Nourse Rogers Memorial Veterans Hospital (John Wells)
  200 Springs Road, Bedford, MA 01730
- Edward Hines, Jr. VA Medical Center (Salvador Gutierrez)
  5000 South 5th Avenue, Hines, IL 60141
- Fayetteville VA Medical Center (Gretchen Gibson)
  1100 N College Ave, Fayetteville, AR 72703
- VA Health Care Upstate New York (Laurence Kaminsky)
  113 Holland Avenue Albany NY 12208
- New Mexico VA Health Care System (Gerardo Villareal)
  1501 San Pedro Drive, S.E.Albuquerque, NM 87108
- VA Boston Healthcare System (Scott Kinlay)
  150 S. Huntington Avenue, Boston, MA 02130
- VA Western New York Healthcare System (Junzhe Xu)
  3495 Bailey Avenue   Buffalo, NY 14215-1199
- Ralph H. Johnson VA Medical Center (Mark Hamner)
  109 Bee Street, Mental Health Research, Charleston, SC 29401
- Wm. Jennings Bryan Dorn VA Medical Center (Kathlyn Sue Haddock)
  6439 Garners Ferry Road, Columbia, SC 29209
- VA North Texas Health Care System (Sujata Bhushan)
  4500 S. LANCASTER ROAD, DALLAS, TX 75216
- Hampton VA Medical Center (Pran Iruvanti)
  100 Emancipation Drive, Hampton, VA 23667
- Hunter Holmes McGuire VA Medical Center (Michael Godschalk)
  1201 Broad Rock Blvd., Richmond, VA 23249
- Iowa City VA Health Care System (Zuhair Ballas)
  601 Highway 6 West, Iowa City, IA 52246-2208

- Jack C. Montgomery VA Medical Center (Malcolm Buford)
  1011 Honor Heights Dr., Muskogee, OK 74401
- James A. Haley Veterans' Hospital (Stephen Mastorides)
  13000 Bruce B. Downs Blvd., Tampa, FL 33612
- Louisville VA Medical Center (Jon Klein)
  800 Zorn Avenue, Louisville, KY 40206
- Manchester VA Medical Center (Nora Ratcliffe)
  718 Smyth Road, Manchester, NH 03104

- Miami VA Health Care System (Hermes Florez)
  1201 NW 16th Street, 11 GRC, Miami FL 33125
- Michael E. DeBakey VA Medical Center (Alan Swann)
  2002 Holcombe Blvd. Houston TX 77030
- Minneapolis VA Health Care System (Maureen Murdoch)
  One Veterans Drive Minneapolis MN 55417
- N. FL/S. GA Veterans Health System (Peruvemba Sriram)
  1601 SW Archer Road, Gainesville, FL 32608
- Northport VA Medical Center (Shing Shing Yeh)
  79 Middleville Road, Northport, NY 11768
- Overton Brooks VA Medical Center (Ronald Washburn)
  510 East Stoner Ave, Shreveport, LA 71101
- Philadelphia VA Medical Center (Darshana Jhala)
  3900 Woodland Avenue, Philadelphia, PA 19104
- Phoenix VA Health Care System (Samuel Aguayo)
  650 E. Indian School Road, Phoenix, AZ 85012
- Portland VA Medical Center (David Cohen)
  3710 SW U.S. Veterans Hospital Road, Portland, OR 97239
- Providence VA Medical Center (Satish Sharma)
  830 Chalkstone Avenue, Providence, RI 02908
- Richard Roudebush VA Medical Center (John Callaghan)
  1481 West 10th Street, Indianapolis, IN 46202
- Salem VA Medical Center (Kris Ann Oursler)
  1970 Roanoke Blvd.,Salem, VA 24153
- San Francisco VA Health Care System (Mary Whooley)
  4150 Clement Street, San Francisco, CA 94121
- South Texas Veterans Health Care System (Sunil Ahuja)
  7400 Merton Minter Boulevard, San Antonio, TX 78229
- Southeast Louisiana Veterans Health Care System (Amparo Gutierrez)
  2400 Canal Street, New Orleans, LA 70119
- Southern Arizona VA Health Care System (Ronald Schifman)
  3601 S 6th Ave, Tucson, AZ 85723
- Sioux Falls VA Health Care System (Jennifer Greco)
  2501 W 22nd St, Sioux Falls, SD 57105
- St. Louis VA Health Care System (Michael Rauchman)
  915 North Grand Blvd., St. Louis, MO 63106
- Syracuse VA Medical Center (Richard Servatius)
  800 Irving Avenue, Syracuse, NY 13210
- VA Eastern Kansas Health Care System (Mary Oehlert)
  4101 S 4th Street Trafficway, Leavenworth, KS 66048
- VA Greater Los Angeles Health Care System (Agnes Wallbom)
  11301 Wilshire Blvd Los Angeles, CA 90073
- VA Loma Linda Healthcare System (Ronald Fernando)
  11201 Benton Street, Loma Linda, CA 92357
- VA Long Beach Healthcare System (Timothy Morgan)

5901 East 7th Street   Long Beach CA 90822

- VA Maine Healthcare System (Todd Stapley)
  1 VA Center, Augusta, ME 04330
- VA New York Harbor Healthcare System (Scott Sherman)
  423 East 23rd Street   New York, NY 10010
- VA Pacific Islands Health Care System (Gwenevere Anderson)
  459 Patterson Rd, Honolulu, HI 96819
- VA Palo Alto Health Care System (Philip Tsao)
  3801 Miranda Avenue   Palo Alto, CA 94304-1290
- VA Pittsburgh Health Care System (Elif Sonel)
  University Drive, Pittsburgh, PA 15240
- VA Puget Sound Health Care System (Edward Boyko)
  1660 S. Columbian Way Seattle, WA 98108-1597
- VA Salt Lake City Health Care System (Laurence Meyer)
  500 Foothill Drive Salt Lake City, UT 84148
- VA San Diego Healthcare System (Samir Gupta)
  3350 La Jolla Village Drive, San Diego, CA 92161
- VA Southern Nevada Healthcare System (Joseph Fayad)
  6900 North Pecos Road, North Las Vegas, NV 89086
- VA Tennessee Valley Healthcare System (Adriana Hung)
  1310 24th Ave. South Nashville, TN 37212
- Washington DC VA Medical Center (Jack Lichy)
  50 Irving St, Washington, D. C. 20422
- W.G. (Bill) Hefner VA Medical Center (Robin Hurley)
  1601 Brenner Ave, Salisbury, NC 28144
- White River Junction VA Medical Center (Brooks Robey)
  163 Veterans Drive, White River Junction, VT 05009
- William S. Middleton Memorial Veterans Hospital (Robert Striker)
  2500 Overlook Terrace, Madison, WI 53705

References


1. Richards, S. *et al.* Standards and guidelines for the interpretation of sequence variants: a joint consensus recommendation of the American College of Medical Genetics and Genomics and the Association for Molecular Pathology. *Genet Med* **17**, 405-24 (2015).
2. LeMaire, S.A. *et al.* Genome-wide association study identifies a susceptibility locus for thoracic aortic aneurysms and aortic dissections spanning FBN1 at 15q21.1. *Nat Genet* **43**, 996-1000 (2011).
3. Guo, D.C. *et al.* Genetic Variants in LRP1 and ULK4 Are Associated with Acute Aortic Dissections. *Am J Hum Genet* **99**, 762-769 (2016).
4. Roychowdhury, T. *et al.* Regulatory variants in TCF7L2 are associated with thoracic aortic aneurysm. *Am J Hum Genet* **108**, 1578-1589 (2021).
5. The 1000 Genomes Project Consortium. A global reference for human genetic variation. *Nature* **526**, 68-74 (2015).
6. Manichaikul, A. *et al.* Robust relationship inference in genome-wide association studies. *Bioinformatics* **26**, 2867-73 (2010).
7. Fang, H. *et al.* Harmonizing Genetic Ancestry and Self-identified Race/Ethnicity in Genome-wide Association Studies. *Am J Hum Genet* **105**, 763-772 (2019).
8. Delaneau, O., Zagury, J.F., Robinson, M.R., Marchini, J.L. & Dermitzakis, E.T. Accurate, scalable and integrative haplotype estimation. *Nat Commun* **10**, 5436 (2019).
9. Howie, B., Fuchsberger, C., Stephens, M., Marchini, J. & Abecasis, G.R. Fast and accurate genotype imputation in genome-wide association studies through pre-phasing. *Nat Genet* **44**, 955-9 (2012).
10. Galinsky, K.J. *et al.* Fast Principal-Component Analysis Reveals Convergent Evolution of ADH1B in Europe and East Asia. *Am J Hum Genet* **98**, 456-472 (2016).
11. Winkler, T.W. *et al.* Quality control and conduct of genome-wide association meta-analyses. *Nat Protoc* **9**, 1192-212 (2014).
12. Mbatchou, J. *et al.* Computationally efficient whole-genome regression for quantitative and binary traits. *Nat Genet* **53**, 1097-1103 (2021).
13. Gusev, A. *et al.* Integrative approaches for large-scale transcriptome-wide association studies. *Nat Genet* **48**, 245-52 (2016).
14. Mancuso, N. *et al.* Probabilistic fine-mapping of transcriptome-wide association studies. *Nat Genet* **51**, 675-682 (2019).
15. GTEx Consortium. Genetic effects on gene expression across human tissues. *Nature* **550**, 204-213 (2017).
16. Hormozdiari, F., Kichaev, G., Yang, W.Y., Pasaniuc, B. & Eskin, E. Identification of causal genes for complex traits. *Bioinformatics* **31**, i206-13 (2015).
17. Guo, H. *et al.* Integration of disease association and eQTL data using a Bayesian colocalisation approach highlights six candidate causal genes in immune-mediated diseases. *Hum Mol Genet* **24**, 3305-13 (2015).
18. Zuber, V., Colijn, J.M., Klaver, C. & Burgess, S. Selecting likely causal risk factors from high-throughput experiments using multivariable Mendelian randomization. *Nat Commun* **11**, 29 (2020).
19. Pan-UKB team. https://pan.ukbb.broadinstitute.org. (2020).
20. Nyholt, D.R. A simple correction for multiple testing for single-nucleotide polymorphisms in linkage disequilibrium with each other. *Am J Hum Genet* **74**, 765-9 (2004).

21. Zuber, V. *et al.* High-throughput multivariable Mendelian randomization analysis prioritizes apolipoprotein B as key lipid risk factor for coronary artery disease. *Int J Epidemiol* **50**, 893-901 (2021).
22. Bycroft, C. *et al.* The UK Biobank resource with deep phenotyping and genomic data. *Nature* **562**, 203-209 (2018).
23. Smoller, J.W. *et al.* An eMERGE Clinical Center at Partners Personalized Medicine. *J Pers Med* **6**(2016).
