# Supplementary material for: Genome-wide association study of thoracic aortic aneurysm and dissection in the Million Veteran Program
Source: Nat Genet. 2023 Jun 12;55(7):1106–15. doi: 10.1038/s41588-023-01420-z (PMC10335930; doi:10.1038/s41588-023-01420-z)
Supplement: Supplementary file 2 — Reporting Summary [file 41588_2023_1420_MOESM2_ESM.pdf]

nature research

Corresponding author(s): Derek Klarin, MD
Scott Damrauer, MD

Last updated by author(s): Dec 17, 2022

# Reporting Summary

Nature Research wishes to improve the reproducibility of the work that we publish. This form provides structure for consistency and transparency in reporting. For further information on Nature Research policies, see our Editorial Policies and the Editorial Policy Checklist.

## Statistics

For all statistical analyses, confirm that the following items are present in the figure legend, table legend, main text, or Methods section.

| n/a | Confirmed | |
|---|---|---|
| ☐ | ☒ | The exact sample size (*n*) for each experimental group/condition, given as a discrete number and unit of measurement |
| ☐ | ☒ | A statement on whether measurements were taken from distinct samples or whether the same sample was measured repeatedly |
| ☐ | ☒ | The statistical test(s) used AND whether they are one- or two-sided<br>*Only common tests should be described solely by name; describe more complex techniques in the Methods section.* |
| ☐ | ☒ | A description of all covariates tested |
| ☐ | ☒ | A description of any assumptions or corrections, such as tests of normality and adjustment for multiple comparisons |
| ☐ | ☒ | A full description of the statistical parameters including central tendency (e.g. means) or other basic estimates (e.g. regression coefficient) AND variation (e.g. standard deviation) or associated estimates of uncertainty (e.g. confidence intervals) |
| ☐ | ☒ | For null hypothesis testing, the test statistic (e.g. *F*, *t*, *r*) with confidence intervals, effect sizes, degrees of freedom and *P* value noted<br>*Give P values as exact values whenever suitable.* |
| ☐ | ☒ | For Bayesian analysis, information on the choice of priors and Markov chain Monte Carlo settings |
| ☐ | ☒ | For hierarchical and complex designs, identification of the appropriate level for tests and full reporting of outcomes |
| ☒ | ☐ | Estimates of effect sizes (e.g. Cohen's *d*, Pearson's *r*), indicating how they were calculated |

*Our web collection on statistics for biologists contains articles on many of the points above.*

## Software and code

Policy information about availability of computer code

| | |
|---|---|
| Data collection | Phenotypic data was collected from the electronic health record and genetic data using the Million Veteran Program (MVP) Axiom array. All data was collated using R-4.1 as documented in the online methods. |
| Data analysis | Data was collected using the EasyQC package and REGENIE v2 software program as outlined in the online methods. Additional software used for analysis include the Coloc R package, FGSEA R Package, FOCUS/TWAS v0.5, Eigensoft v6, PRSCSx v1.0, DENTIST v1.1, GCTA v1.93, Seurat v4. Clear code for analysis is available at their associated website as standard pipelines were implemented. Any code for use in the above software will be provided at reasonable request. |

For manuscripts utilizing custom algorithms or software that are central to the research but not yet described in published literature, software must be made available to editors and reviewers. We strongly encourage code deposition in a community repository (e.g. GitHub). See the Nature Research guidelines for submitting code & software for further information.

## Data

Policy information about availability of data

All manuscripts must include a data availability statement. This statement should provide the following information, where applicable:

- Accession codes, unique identifiers, or web links for publicly available datasets
- A list of figures that have associated raw data
- A description of any restrictions on data availability

The full summary level association data from the MVP TAAD discovery analysis from this manuscript are available through dbGAP, accession code phs001672.v2.p1. UK Biobank individual-level data are available for request by application (https://www.ukbiobank.ac.uk). TAA GWAS summary statistics from CHIP/MGI are available here: http://csg.sph.umich.edu/willer/public/TAA2021/. Individual-level MassGeneral Biobank data and Penn Medicine Biobank data are available from https://personalizedmedicine.partners.org/Biobank/Default.aspx and https://pmbb.med.upenn.edu/, but restrictions apply to the availability of these data, which were

used under IRB approval for the current study, and so are not publicly available. Requests for the use of individual level data from HUNT must be approved by the K.G. Jebsen Center for Genetic Epidemiology at NTNU. Applications are sent to HUNT and then discussed with the center. All single cell/single nucleus RNA sequencing data were previously made publicly available at the Gene Expression Omnibus (GEO) and can be accessed at GSE155468 or from the Broad Institute Single Cell Portal (https://singlecell.broadinstitute.org/single_cell).

# Field-specific reporting

Please select the one below that is the best fit for your research. If you are not sure, read the appropriate sections before making your selection.

☒ Life sciences ☐ Behavioural & social sciences ☐ Ecological, evolutionary & environmental sciences

For a reference copy of the document with all sections, see nature.com/documents/nr-reporting-summary-flat.pdf

# Life sciences study design

All studies must disclose on these points even when the disclosure is negative.

| | |
|---|---|
| Sample size | All samples available of three ancestries (European, African, Hispanic) were used for analysis (after quality control). Sample size was determined based on genetic data available from MVP. Participants were excluded if they failed to meet case or control definitions. |
| Data exclusions | Data were excluded if they did not pass our QC metrics, or if they did not fall within the three main ancestries used for analysis |
| Replication | Replication was performed using data from 6 external cohorts and were successfully replicated for 17 of 22 novel genome-wide significant loci |
| Randomization | Randomization is not relevant for this binary trait GWAS study which is a retrospective analysis of TAAD cases and controls. |
| Blinding | Randomization is not relevant for this binary trait GWAS study which is a retrospective analysis of TAAD cases and controls. |

# Reporting for specific materials, systems and methods

We require information from authors about some types of materials, experimental systems and methods used in many studies. Here, indicate whether each material, system or method listed is relevant to your study. If you are not sure if a list item applies to your research, read the appropriate section before selecting a response.

## Materials & experimental systems

| n/a | Involved in the study | |
|---|---|---|
| ☒ | ☐ | Antibodies |
| ☒ | ☐ | Eukaryotic cell lines |
| ☒ | ☐ | Palaeontology and archaeology |
| ☒ | ☐ | Animals and other organisms |
| ☐ | ☒ | Human research participants |
| ☒ | ☐ | Clinical data |
| ☒ | ☐ | Dual use research of concern |

## Methods

| n/a | Involved in the study | |
|---|---|---|
| ☒ | ☐ | ChIP-seq |
| ☒ | ☐ | Flow cytometry |
| ☒ | ☐ | MRI-based neuroimaging |

## Human research participants

Policy information about studies involving human research participants

| | |
|---|---|
| Population characteristics | Demographics and participant counts for the European, African, and Hispanic ancestry participants that passed our quality control and were included in the analysis are depicted in Supplementary Table 1 and Supplementary Table 19. |
| Recruitment | Individuals aged 19 to 104 years have been recruited voluntarily from more than 50 VA Medical Centers nationwide for participation in the Million Veteran Program biobank study. Veterans are identified from VA databases and recruited via invitational and appointment mailings. In addition, Veterans are recruited at selected VHA clinical sites. The UK Biobank is a population-based cohort of approximately 500,000 participants recruited from 2006-2010 with existing genomic and longitudinal phenotypic data and median 10-year follow-up. Baseline assessments were conducted at 22 assessment centres across the UK with sample collections including blood-derived DNA. Use of the data was facilitated through UK Biobank Application 7089. The Cardiovascular Health Improvement Project (CHIP) is a cohort of individuals treated at Michigan Medicine with linked genotype, EHR, and family history data. The Michigan Genomics Initiative (MGI) is a hospital-based cohort with linked genotype and EHR data from participants recruited during pre-surgical encounters at Michigan Medicine. Penn Medicine biobank (PMBB) recruits patients from throughout the University of Pennsylvania Health System for genomic and precision medicine research. Participants actively consent to allow the linkage of biospecimens to their longitudinal EHR. Currently, >60000 participants are enrolled in the PMBB. A further subset of ~23,000 subjects with imputed genotype data was used in this analysis. The MGBB contains genotypic and clinical data from >105,000 patients who consented to broad-based research across 7 regional hospitals and median 3-year follow-up. Baseline phenotypes were ascertained from the electronic medical record and surveys. The Nord-Trøndelag Health Study (HUNT) is a population-based health survey |

conducted in the county of Nord-Trøndelag, Norway, since 1984. Individuals were included at three different time points during approximately 20 years of follow up. Data contributed from this study comprised a GWAS of 765 individuals with sporadic ascending aortic aneurysms or classic aortic dissection of the ascending or descending thoracic aorta (Stanford types A and B, respectively) who presented for treatment at the Texas Medical Center. The diagnosis of TAAD was confirmed by cross-sectional imaging in all subjects and by direct inspection during surgical repair in most subjects. Controls were individuals free of disease, as described in LeMaire et al. The main bias that may be present in the recruitment of these patients is survivorship bias.

| | |
|---|---|
| Ethics oversight | MVP received ethical/study protocol approval by the VA Central Institutional Review Board, the analysis in UK Biobank was approved by a local Institutional Review Board at Partners Healthcare (protocol 2013P001840). The PMBB is approved under IRB protocol# 813913 and supported by Perelman School of Medicine at University of Pennsylvania. Informed consent was obtained for all participants. The use of previously generated summary statistics for CHIP, MGI, HUNT, and University of Texas Health Science Center at Houston was approved by local institutional IRB. In all studies, informed consent was obtained for all participants. |

Note that full information on the approval of the study protocol must also be provided in the manuscript.
